# Supplementary figures and images for: A concerted mechanism involving ACAT and SREBPs by which oxysterols deplete accessible cholesterol to restrict microbial infection
Source: eLife. 2023 Jan 25;12:e83534. doi: 10.7554/eLife.83534 (PMC9925056; doi:10.7554/eLife.83534)

**Figure 1 - Source Blots**

**A**

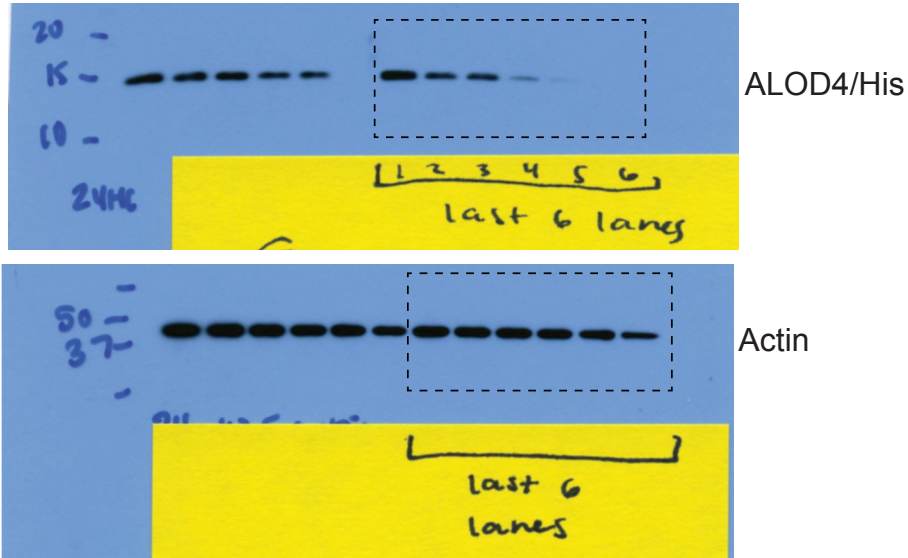

**B**

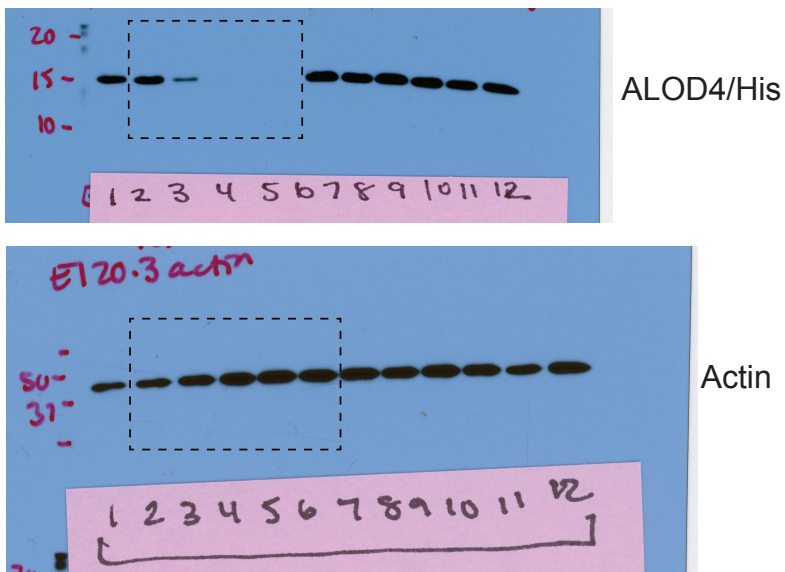

D

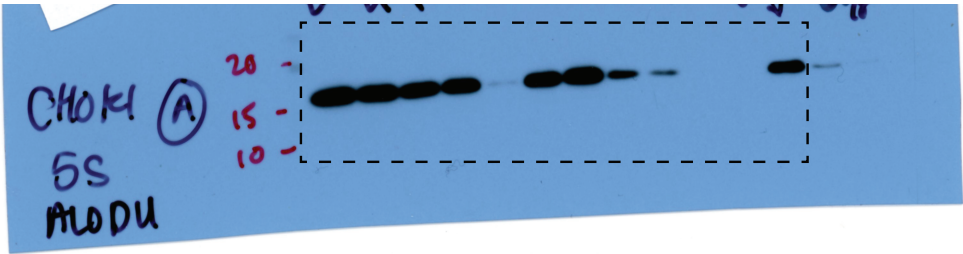

ALOD4/His

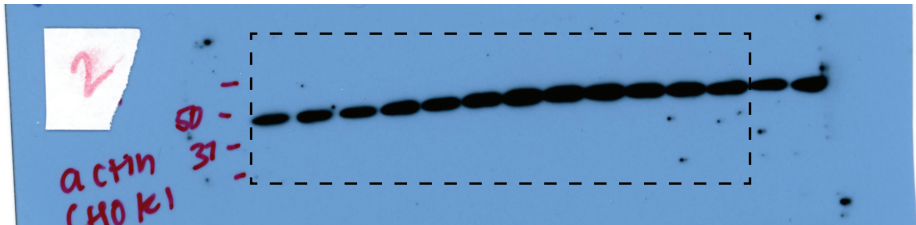

Actin

E

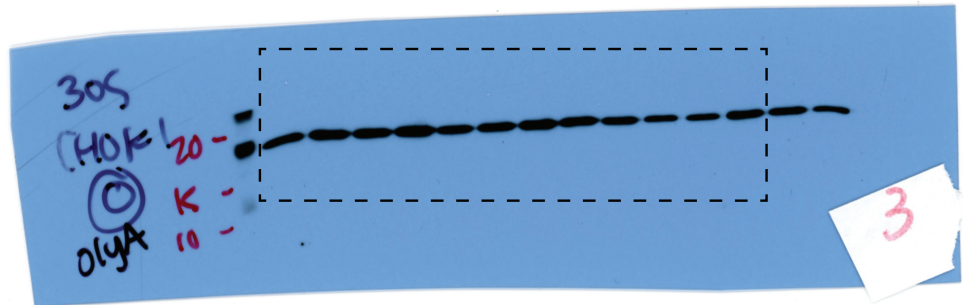

OlyA/His

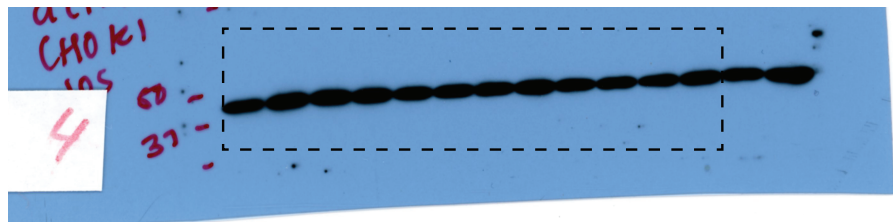

Actin

G

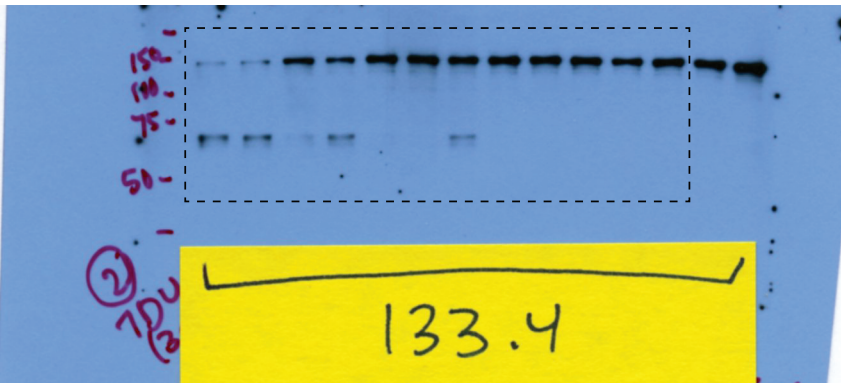

SREBP2/7D4

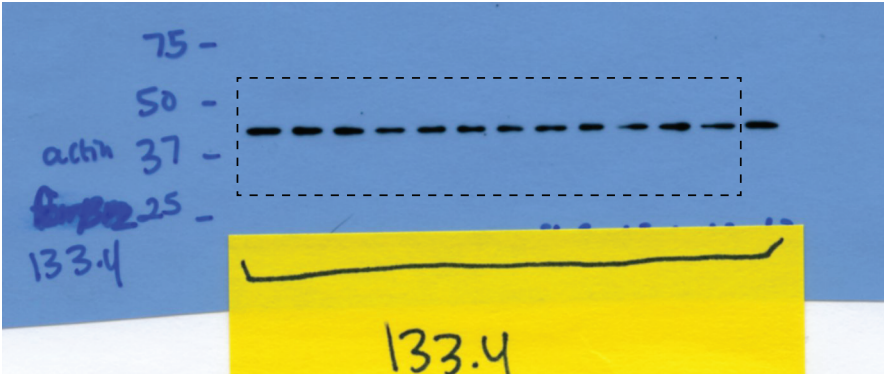

Actin

Supplement: Figure 1—source data 1. [file elife-83534-fig1-data1.zip › Figure 1-source data 1/Figure 1-source data 1.pdf]

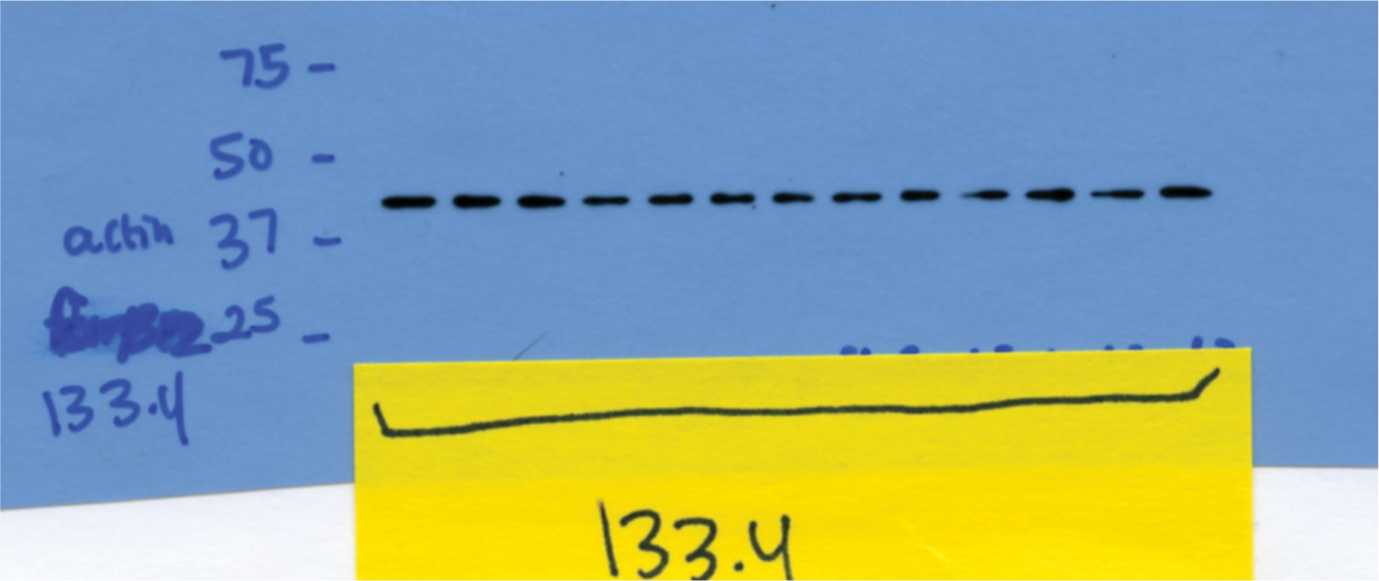

Supplement: Figure 1—source data 1. [file elife-83534-fig1-data1.zip › Figure 1-source data 1/Figure 1G Actin.tif]

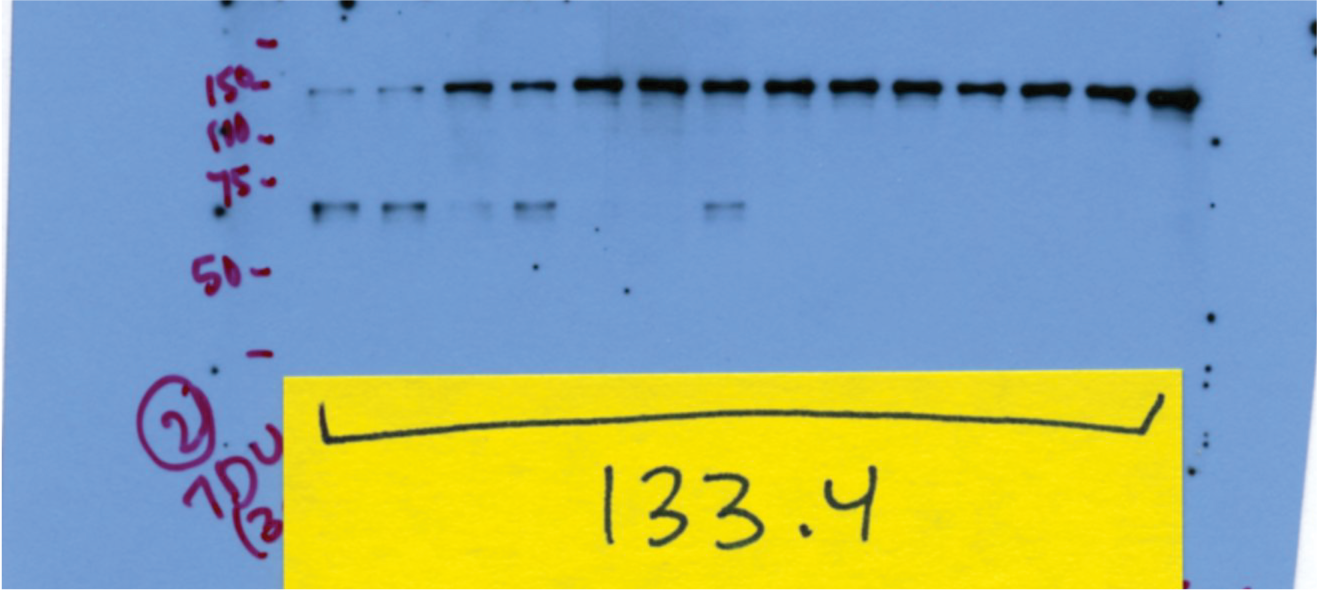

Supplement: Figure 1—source data 1. [file elife-83534-fig1-data1.zip › Figure 1-source data 1/Figure 1G SREBP2.tif]

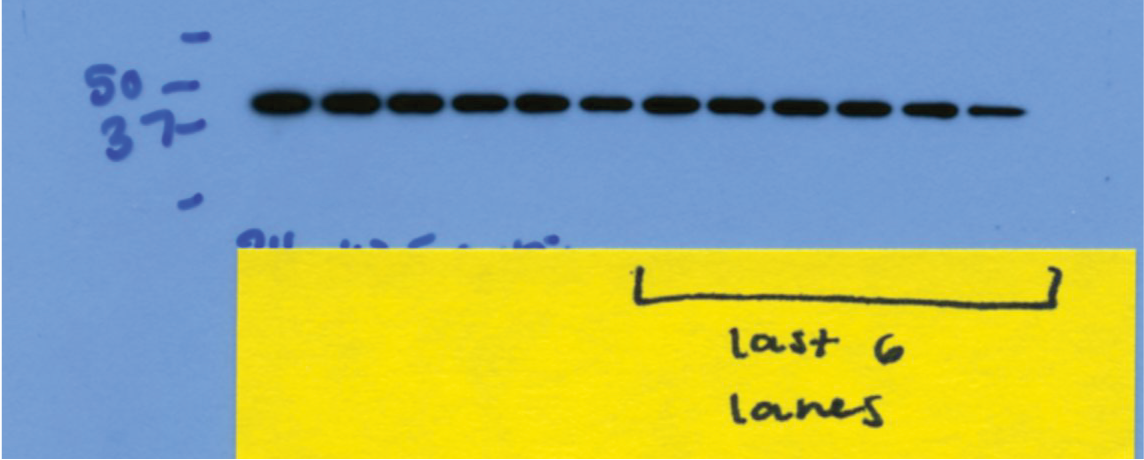

Supplement: Figure 1—source data 1. [file elife-83534-fig1-data1.zip › Figure 1-source data 1/Figure 1A Actin.tif]

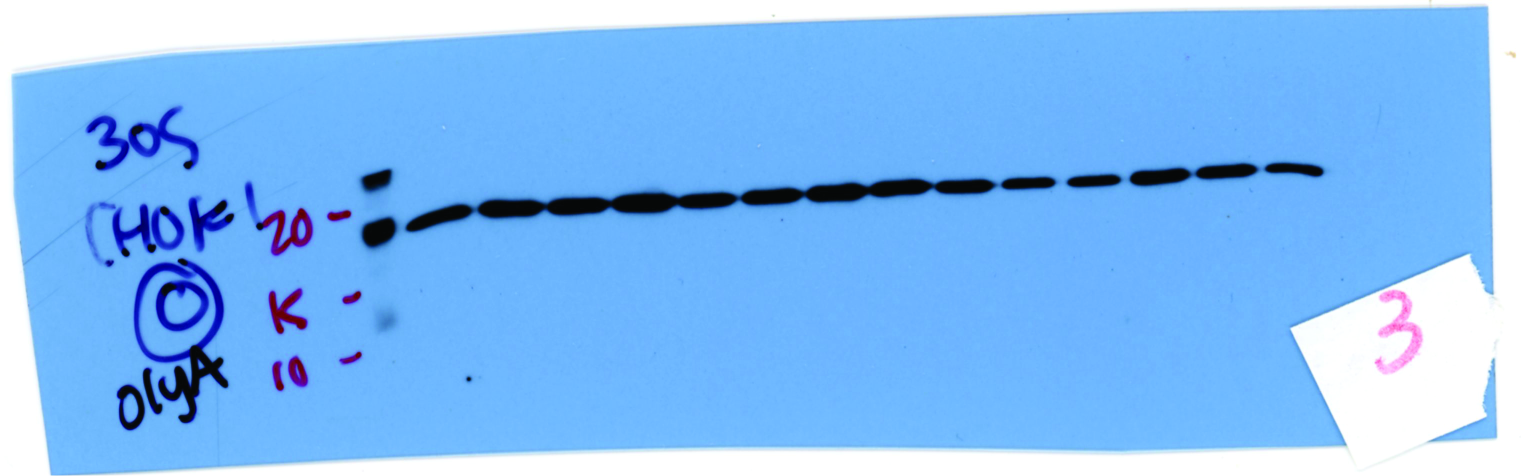

Supplement: Figure 1—source data 1. [file elife-83534-fig1-data1.zip › Figure 1-source data 1/Figure 1E OlyA.tif]

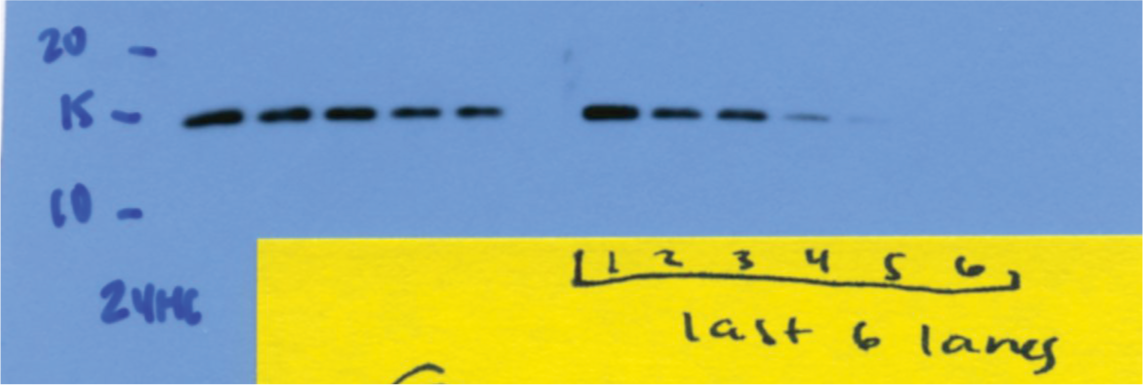

Supplement: Figure 1—source data 1. [file elife-83534-fig1-data1.zip › Figure 1-source data 1/Figure 1A ALOD4.tif]

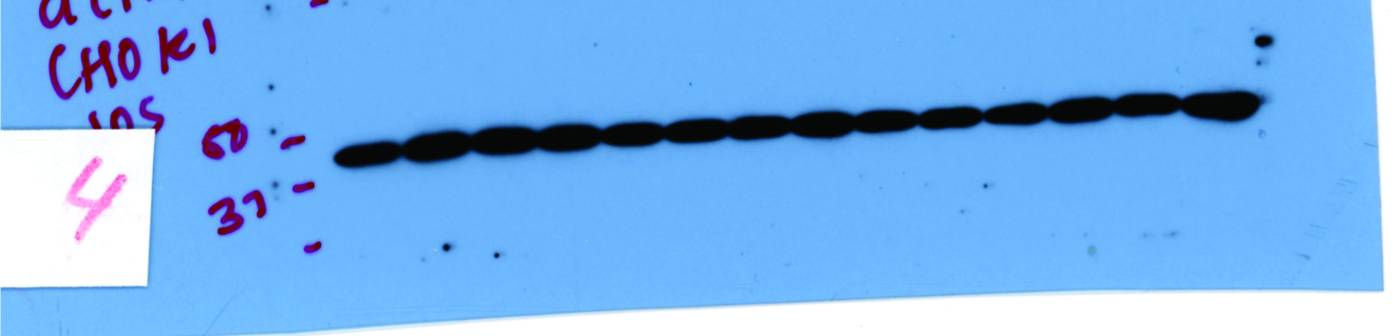

Supplement: Figure 1—source data 1. [file elife-83534-fig1-data1.zip › Figure 1-source data 1/Figure 1E Actin.tif]

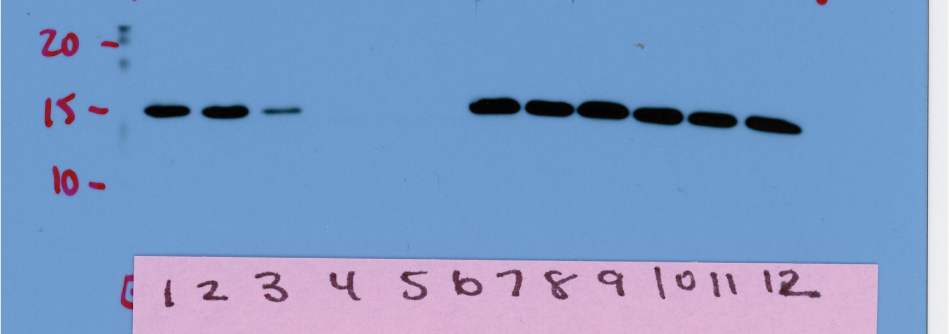

Supplement: Figure 1—source data 1. [file elife-83534-fig1-data1.zip › Figure 1-source data 1/Figure 1B ALOD4.tif]

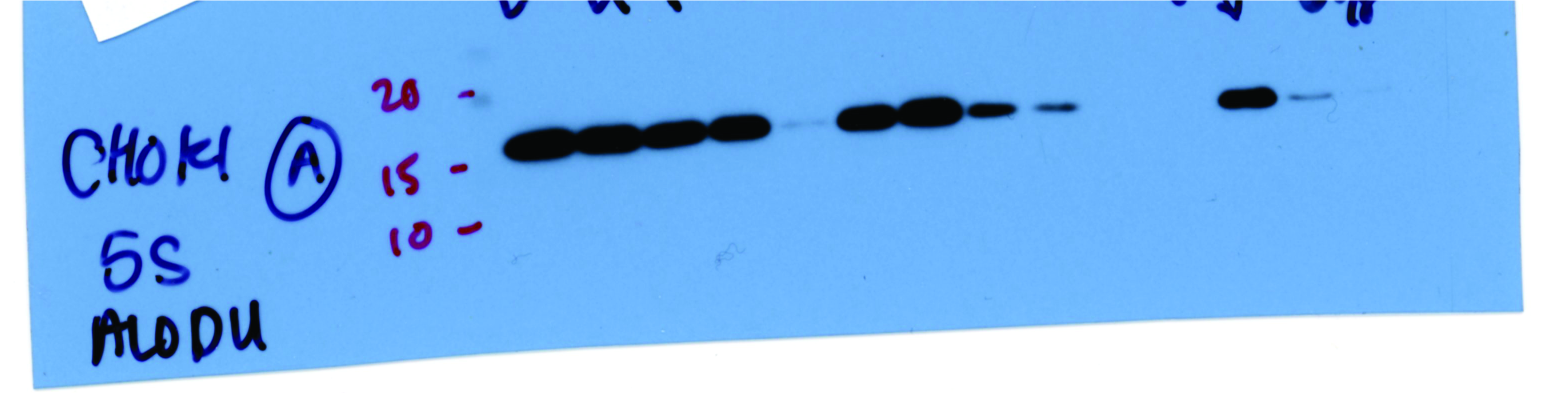

Supplement: Figure 1—source data 1. [file elife-83534-fig1-data1.zip › Figure 1-source data 1/Figure 1D ALOD4.tif]

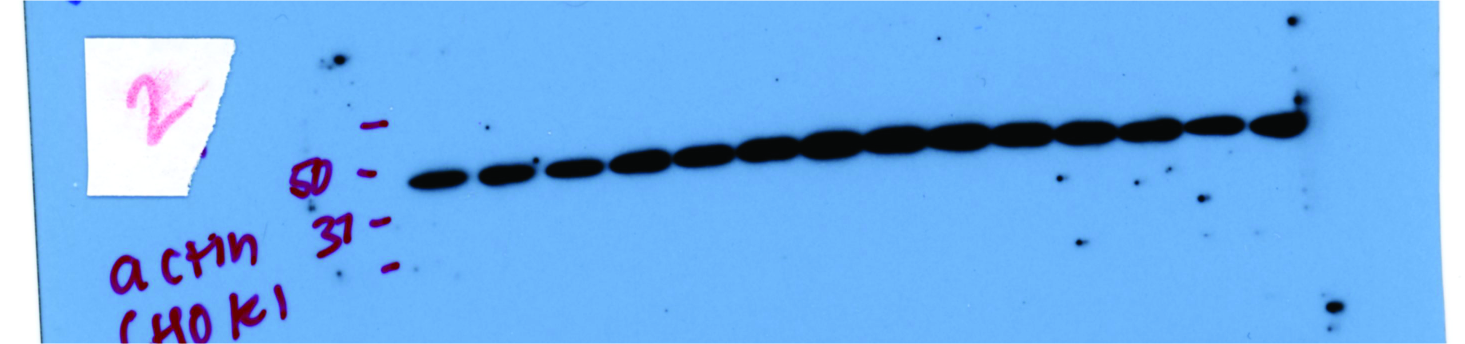

Supplement: Figure 1—source data 1. [file elife-83534-fig1-data1.zip › Figure 1-source data 1/Figure 1D Actin.tif]

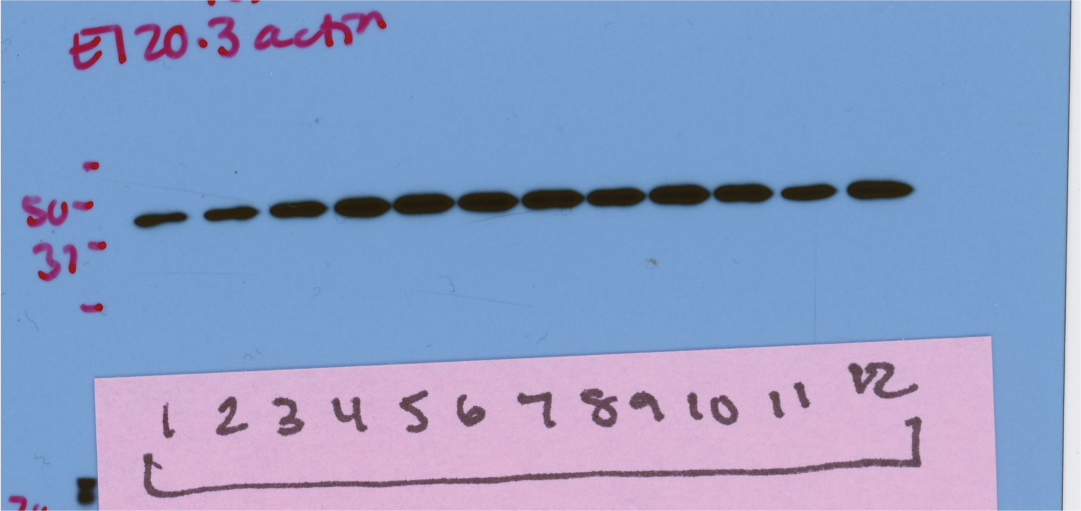

Supplement: Figure 1—source data 1. [file elife-83534-fig1-data1.zip › Figure 1-source data 1/Figure 1B Actin.tif]

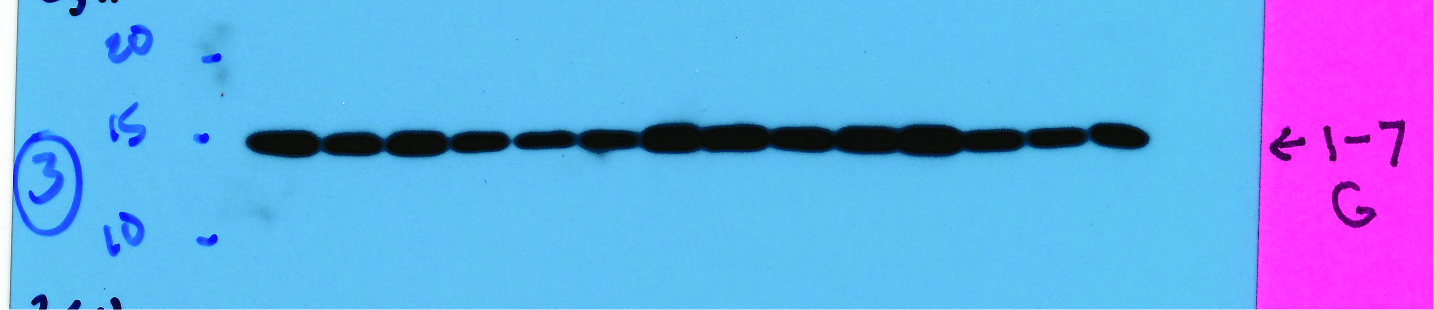

Supplement: Figure 2—source data 1. [file elife-83534-fig2-data1.zip › Figure 2-source data 1/Figure 2A ALOD4 for ACAT1 KO hACAT1(H460A) cells.tif]

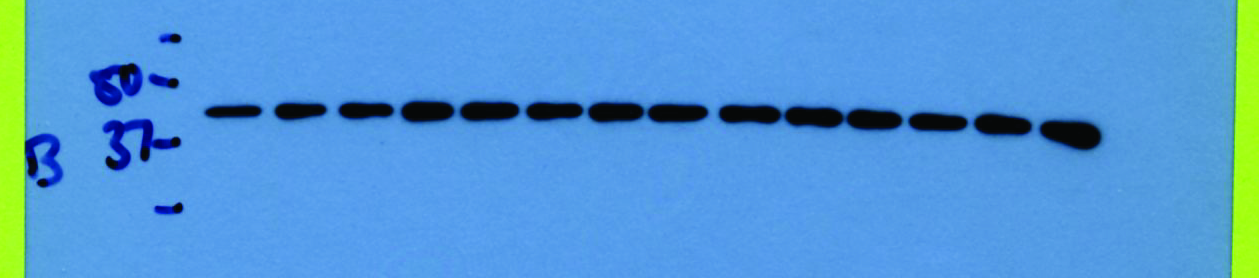

Supplement: Figure 2—source data 1. [file elife-83534-fig2-data1.zip › Figure 2-source data 1/Figure 2D Actin for LXR-deficient ALOD4.tif]

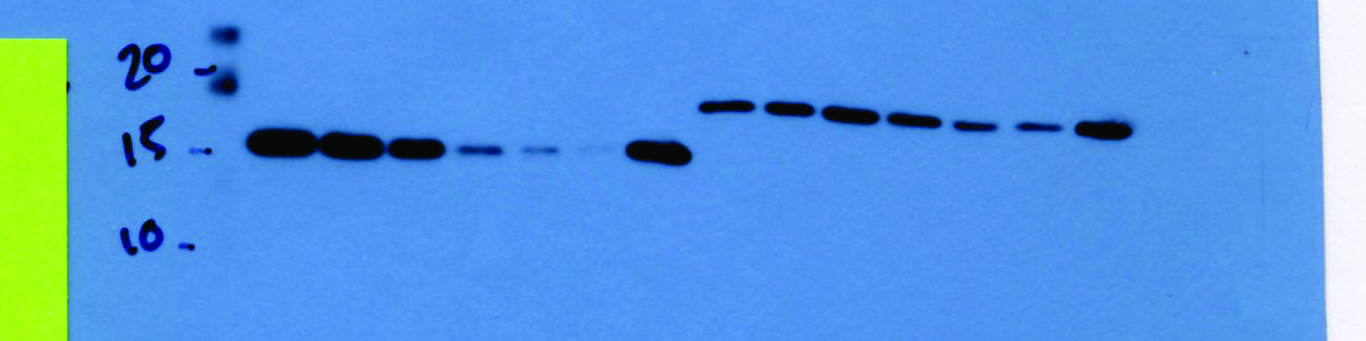

Supplement: Figure 2—source data 1. [file elife-83534-fig2-data1.zip › Figure 2-source data 1/Figure 2D ALOD4 for WT cells.tif]

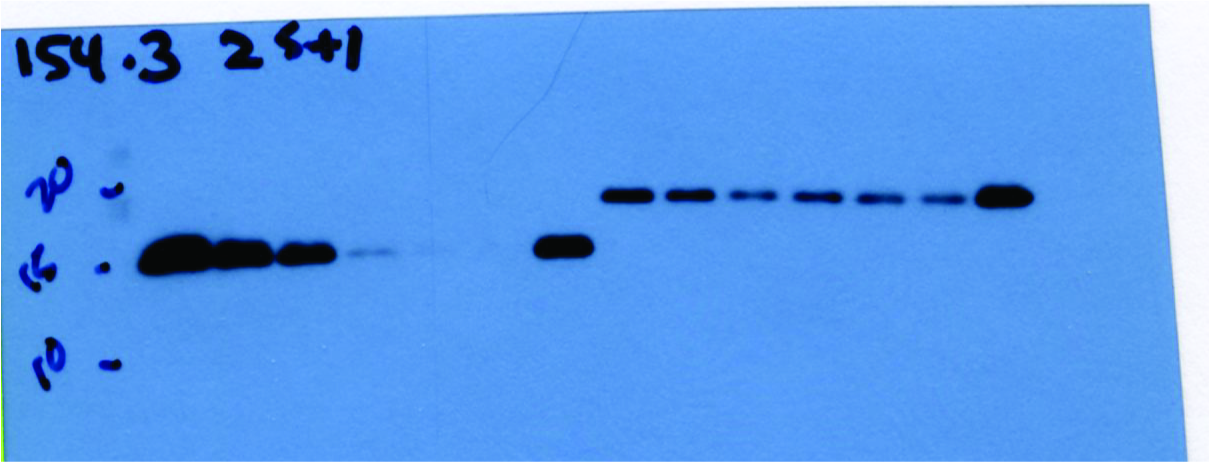

Supplement: Figure 2—source data 1. [file elife-83534-fig2-data1.zip › Figure 2-source data 1/Figure 2C WT ALOD4.tif]

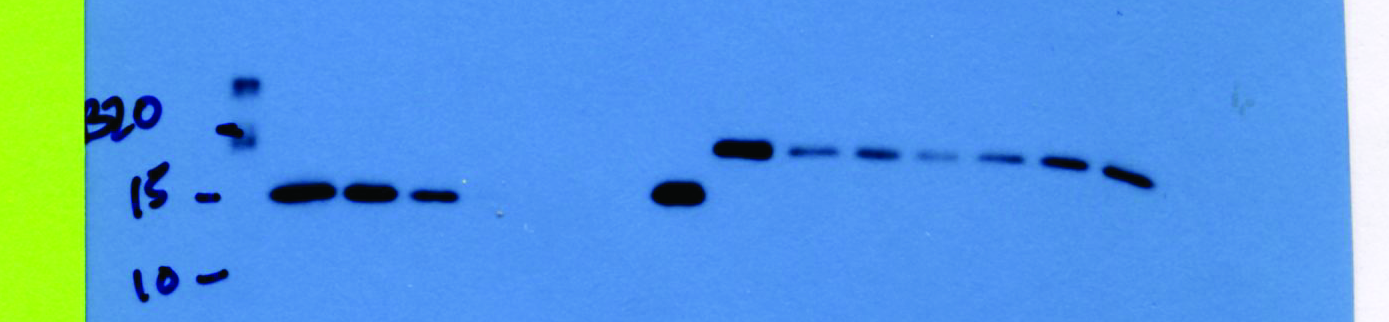

Supplement: Figure 2—source data 1. [file elife-83534-fig2-data1.zip › Figure 2-source data 1/Figure 2D ALOD4 for LXR-deficient cells.tif]

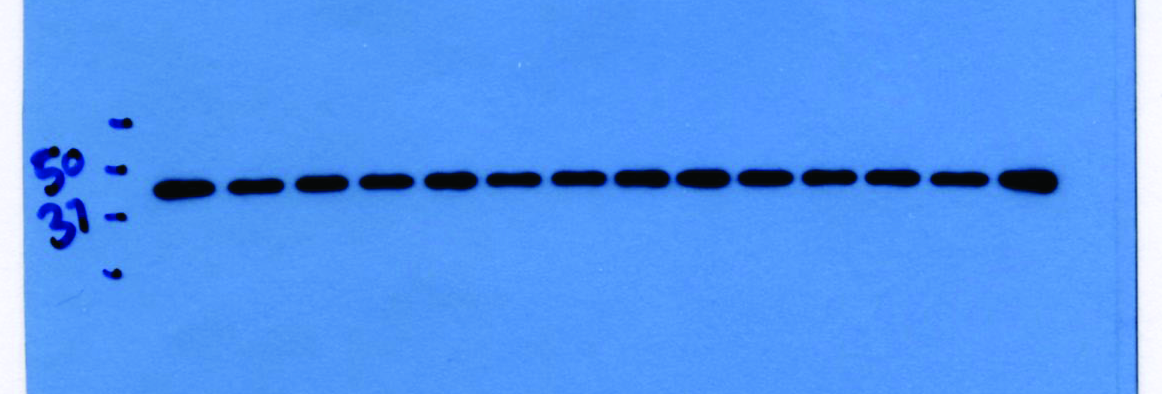

Supplement: Figure 2—source data 1. [file elife-83534-fig2-data1.zip › Figure 2-source data 1/Figure 2C Actin for WT OlyA.tif]

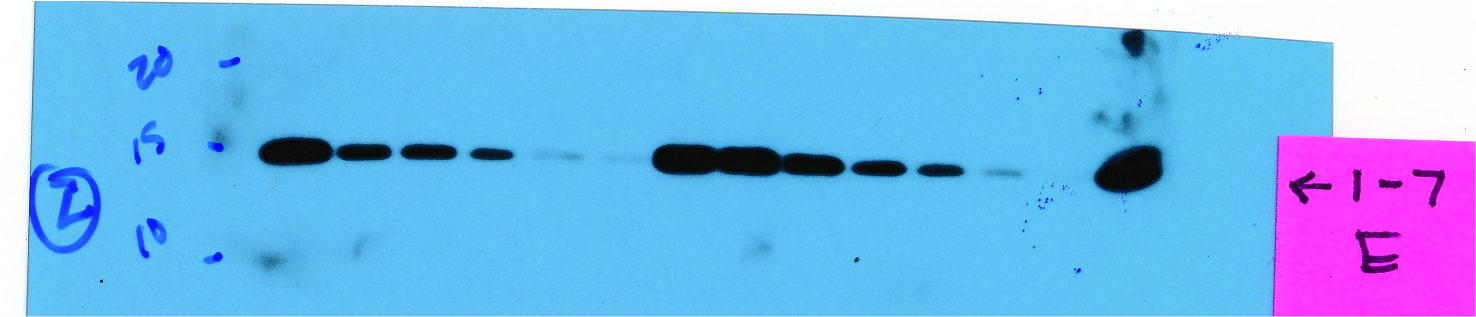

Supplement: Figure 2—source data 1. [file elife-83534-fig2-data1.zip › Figure 2-source data 1/Figure 2A ALOD4 for ACAT1 KO hACAT(WT) cells.tif]

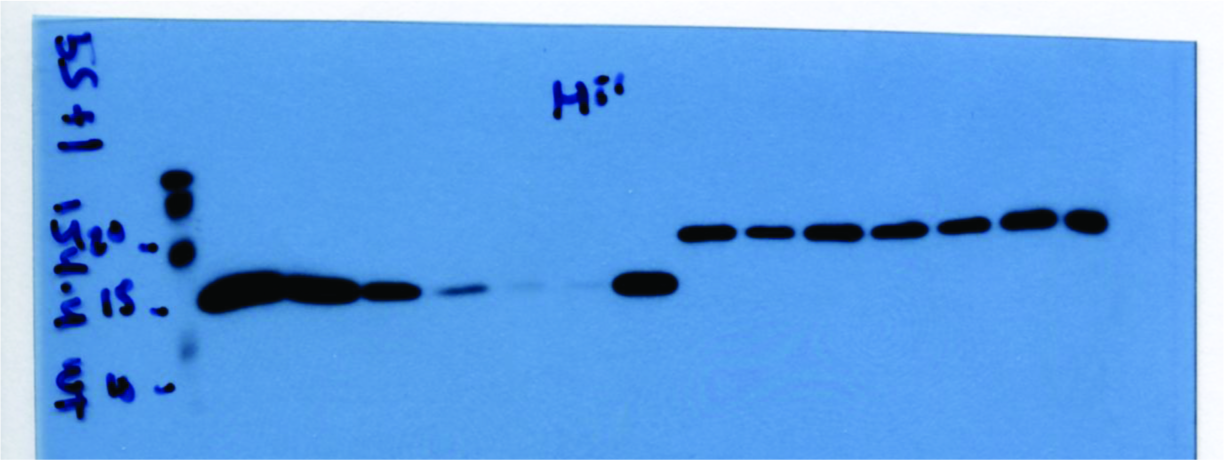

Supplement: Figure 2—source data 1. [file elife-83534-fig2-data1.zip › Figure 2-source data 1/Figure 2C WT OlyA.tif]

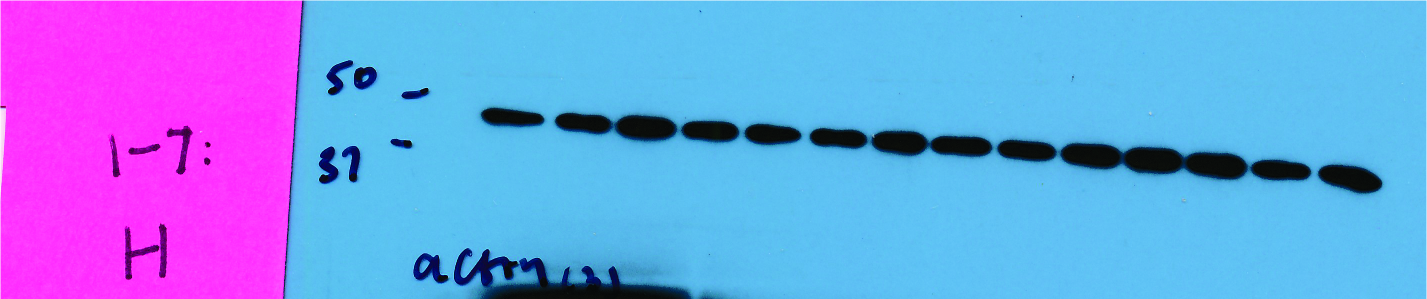

Supplement: Figure 2—source data 1. [file elife-83534-fig2-data1.zip › Figure 2-source data 1/Figure 2A Actin for ACAT1 KOh ACAT1(H460A) cells.tif]

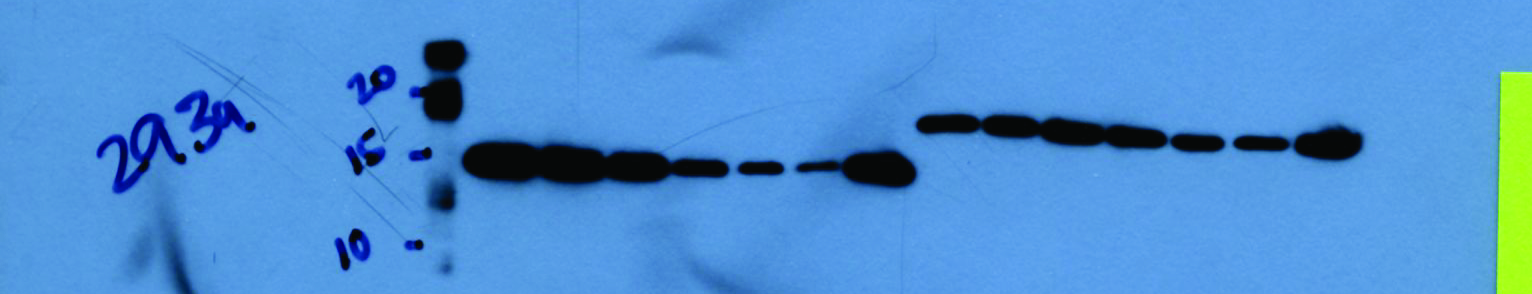

Supplement: Figure 2—source data 1. [file elife-83534-fig2-data1.zip › Figure 2-source data 1/Figure 2D OlyA for WT cells.tif]

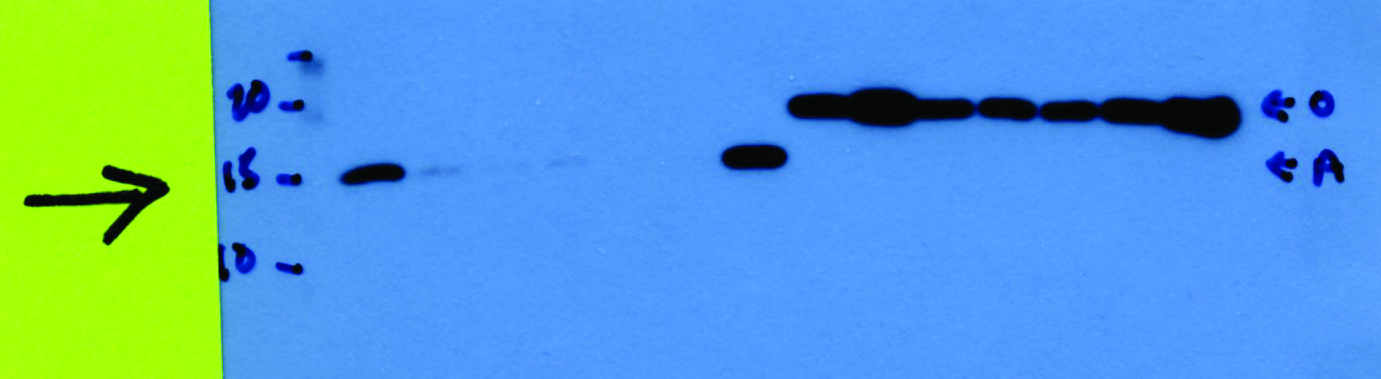

Supplement: Figure 2—source data 1. [file elife-83534-fig2-data1.zip › Figure 2-source data 1/Figure 2C Scap-deficient ALOD4.tif]

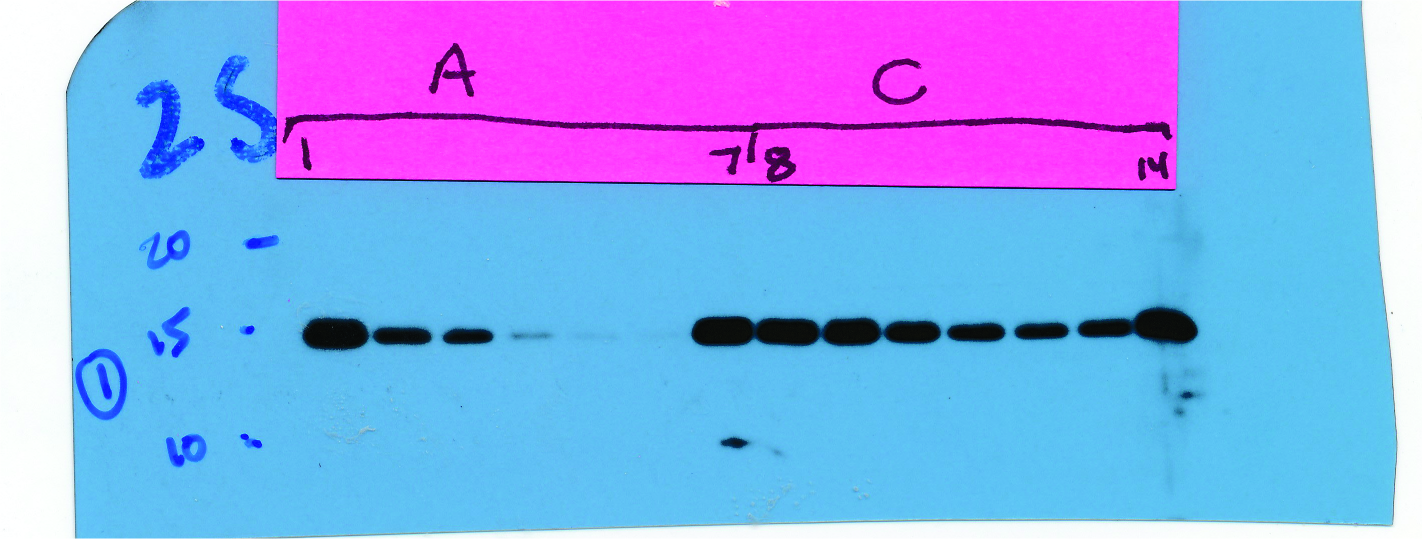

Supplement: Figure 2—source data 1. [file elife-83534-fig2-data1.zip › Figure 2-source data 1/Figure 2A ALOD4 for WT cells.tif]

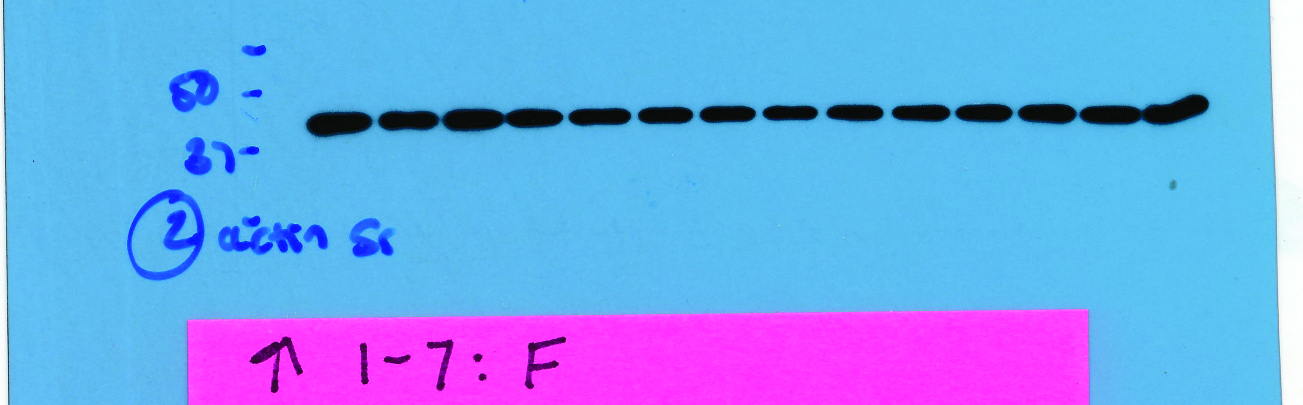

Supplement: Figure 2—source data 1. [file elife-83534-fig2-data1.zip › Figure 2-source data 1/Figure 2A Actin for ACAT1 KO hACAT1(WT) cells.tif]

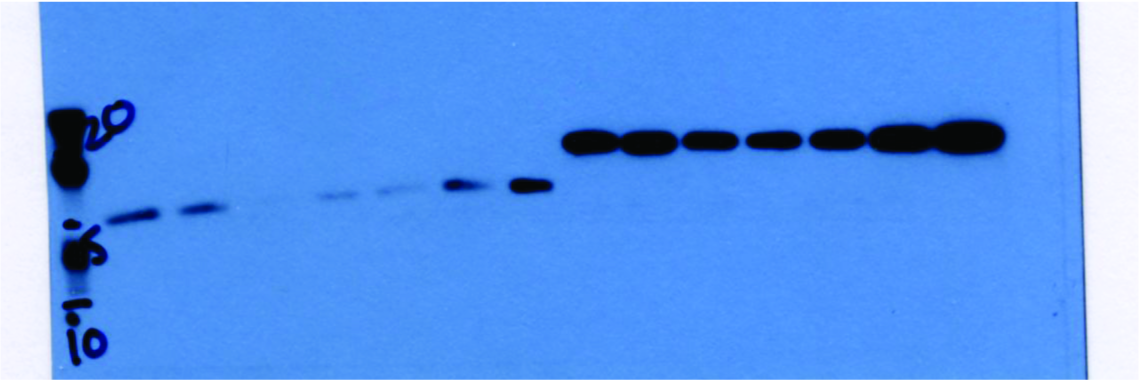

Supplement: Figure 2—source data 1. [file elife-83534-fig2-data1.zip › Figure 2-source data 1/Figure 2C Scap-deficient OlyA.tif]

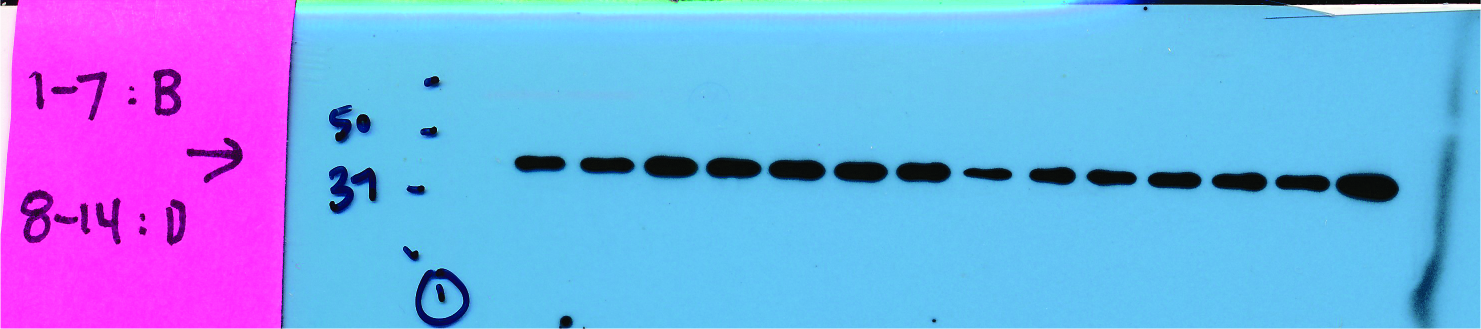

Supplement: Figure 2—source data 1. [file elife-83534-fig2-data1.zip › Figure 2-source data 1/Figure 2A Actin for WT cells.tif]

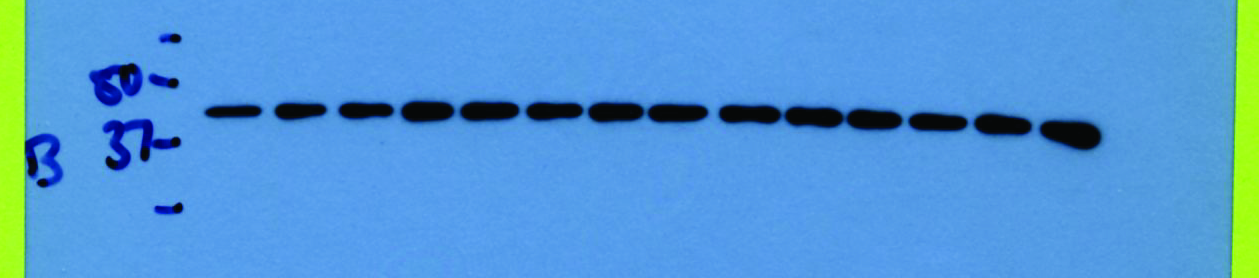

Supplement: Figure 2—source data 1. [file elife-83534-fig2-data1.zip › Figure 2-source data 1/Figure 2D Actin for LXR-deficient OlyA.tif]

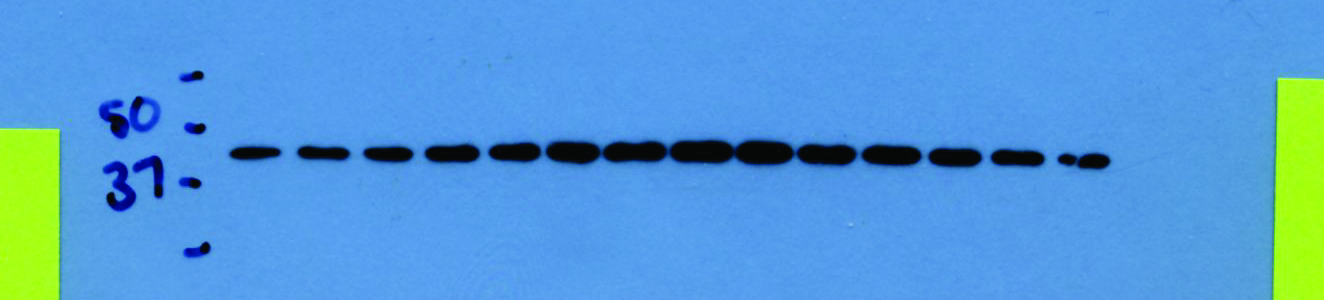

Supplement: Figure 2—source data 1. [file elife-83534-fig2-data1.zip › Figure 2-source data 1/Figure 2D Actin for WT OlyA.tif]

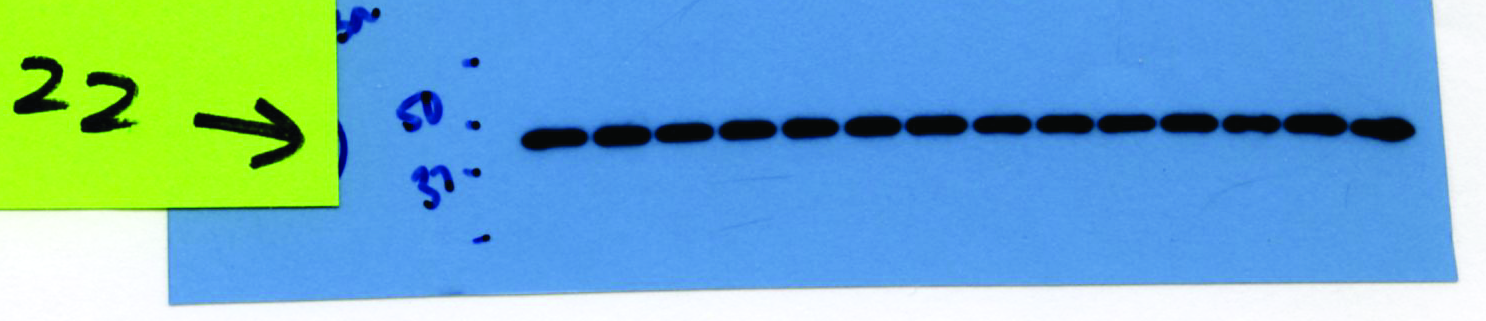

Supplement: Figure 2—source data 1. [file elife-83534-fig2-data1.zip › Figure 2-source data 1/Figure 2C Actin for Scap-deficient ALOD4.tif]

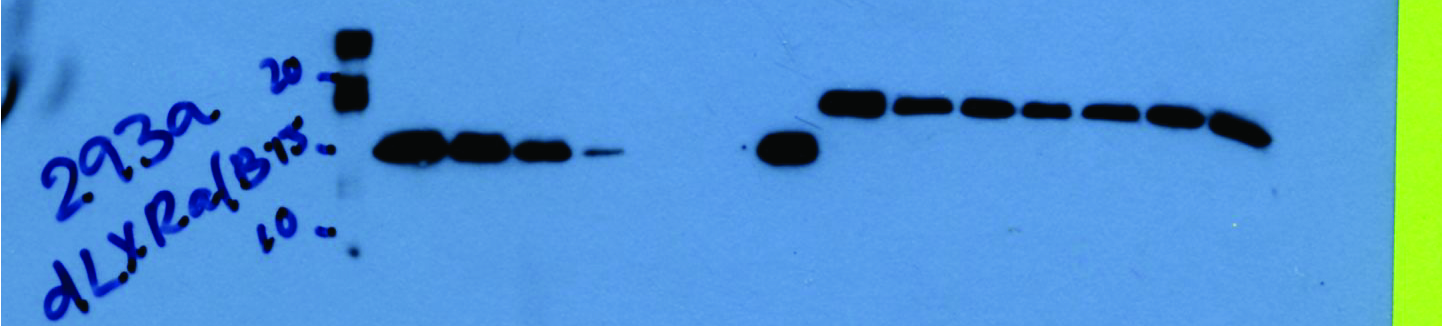

Supplement: Figure 2—source data 1. [file elife-83534-fig2-data1.zip › Figure 2-source data 1/Figure 2D OlyA for LXR-deficient cells.tif]

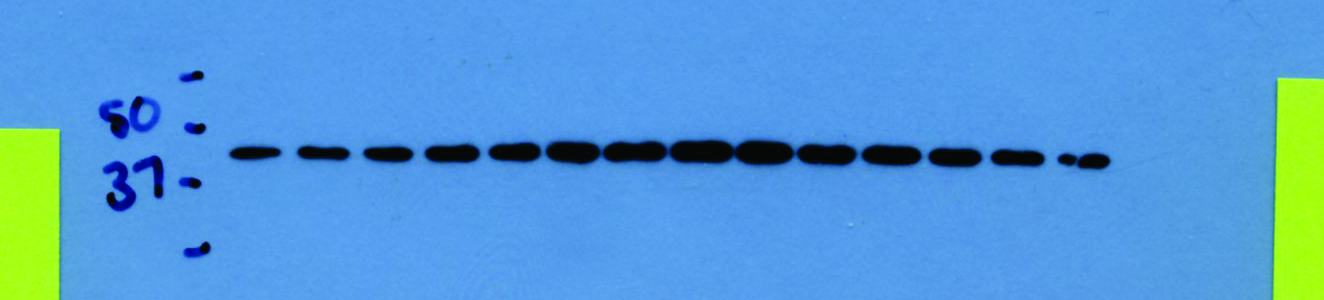

Supplement: Figure 2—source data 1. [file elife-83534-fig2-data1.zip › Figure 2-source data 1/Figure 2D Actin for WT ALOD4.tif]

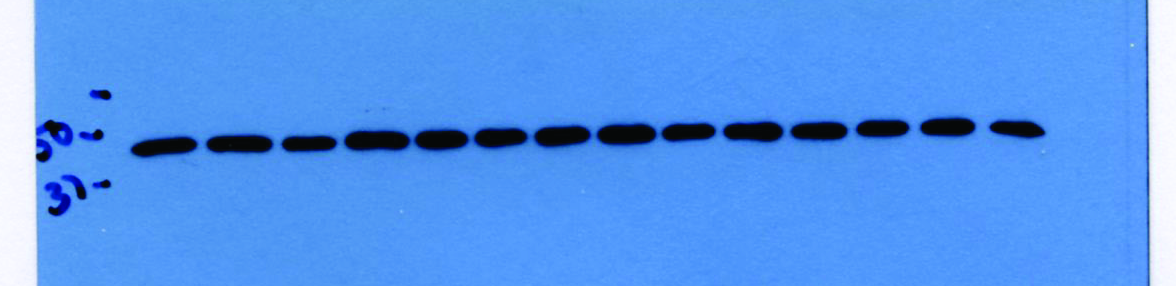

Supplement: Figure 2—source data 1. [file elife-83534-fig2-data1.zip › Figure 2-source data 1/Figure 2C Actin for Scap-deficient OlyA.tif]

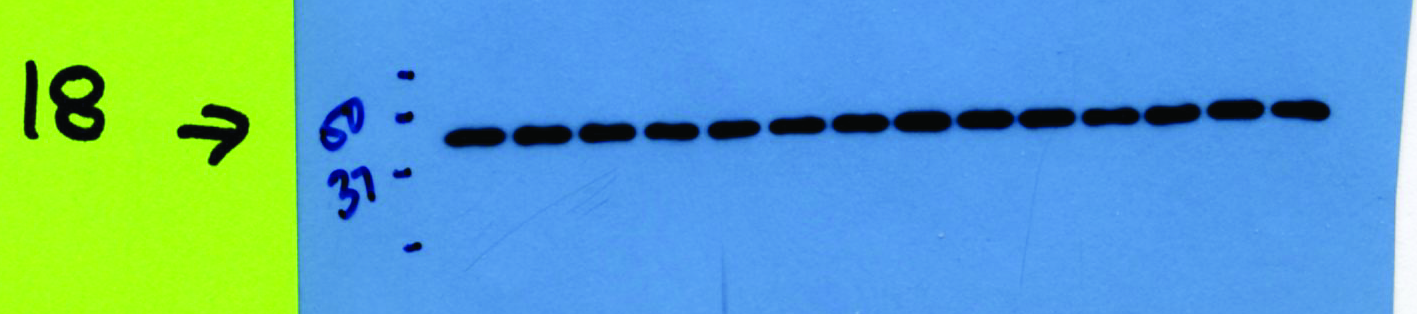

Supplement: Figure 2—source data 1. [file elife-83534-fig2-data1.zip › Figure 2-source data 1/Figure 2C Actin for WT ALOD4.tif]

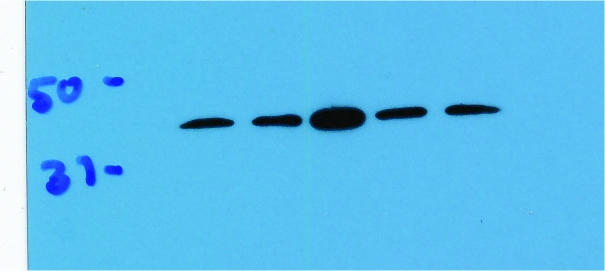

Supplement: Figure 2—figure supplement 1—source data 1. [file elife-83534-fig2-figsupp1-data1.zip › Figure 2-figure supplement 1-source data 1/Figure 2 figure supplement 1C Actin.tif]

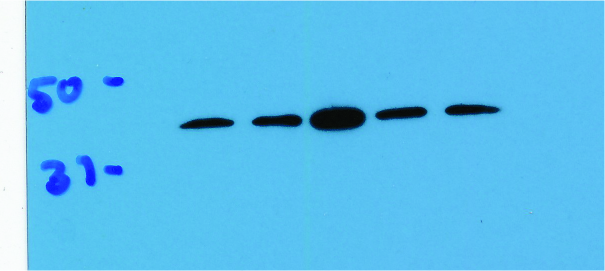

Supplement: Figure 2—figure supplement 1—source data 1. [file elife-83534-fig2-figsupp1-data1.zip › Figure 2-figure supplement 1-source data 1/Figure 2 figure supplement 1B Actin.tif]

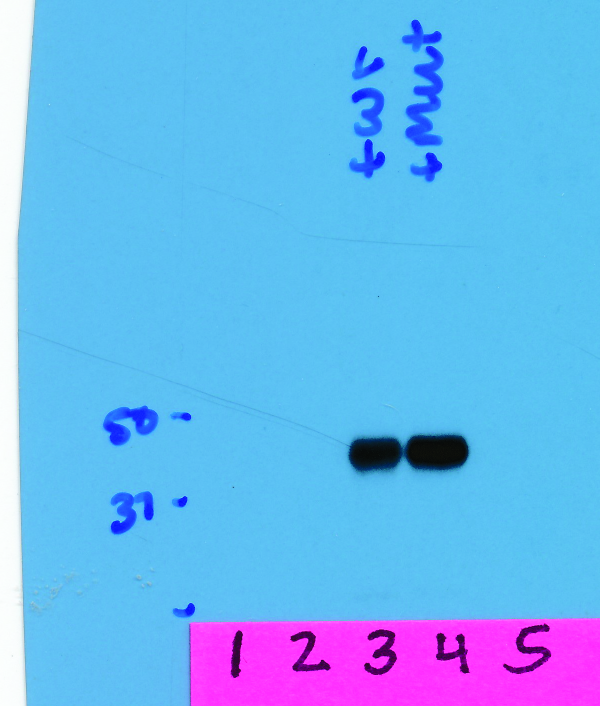

Supplement: Figure 2—figure supplement 1—source data 1. [file elife-83534-fig2-figsupp1-data1.zip › Figure 2-figure supplement 1-source data 1/Figure 2 figure supplement 1C ACAT1.tif]

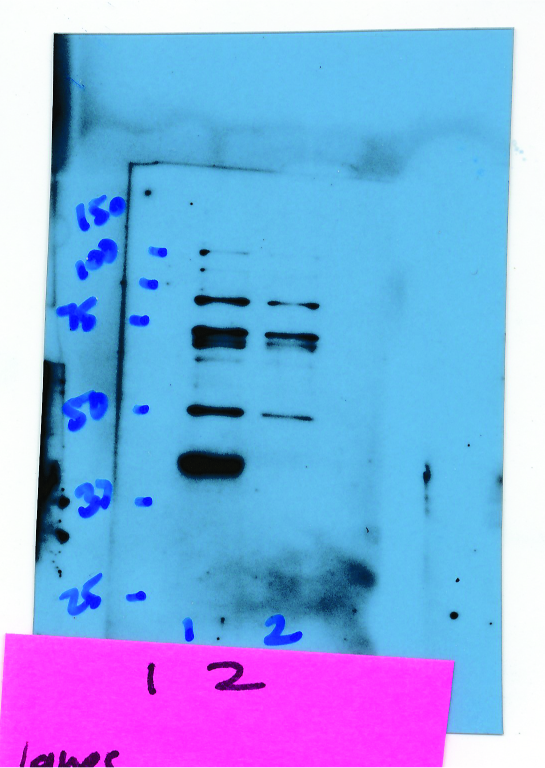

Supplement: Figure 2—figure supplement 1—source data 1. [file elife-83534-fig2-figsupp1-data1.zip › Figure 2-figure supplement 1-source data 1/Figure 2 figure supplement 1B ACAT1.tif]

Figure 2 figure supplement 1 - Source Blots

B

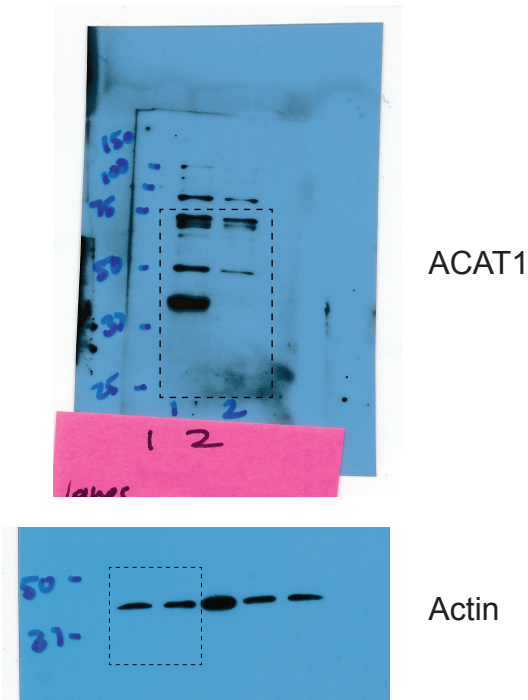

C

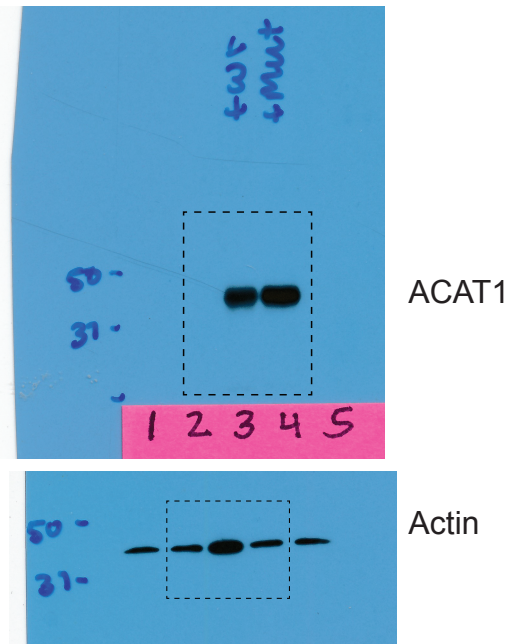

Supplement: Figure 2—figure supplement 1—source data 1. [file elife-83534-fig2-figsupp1-data1.zip › Figure 2-figure supplement 1-source data 1/Figure 2-figure supplement-source data 1.pdf]

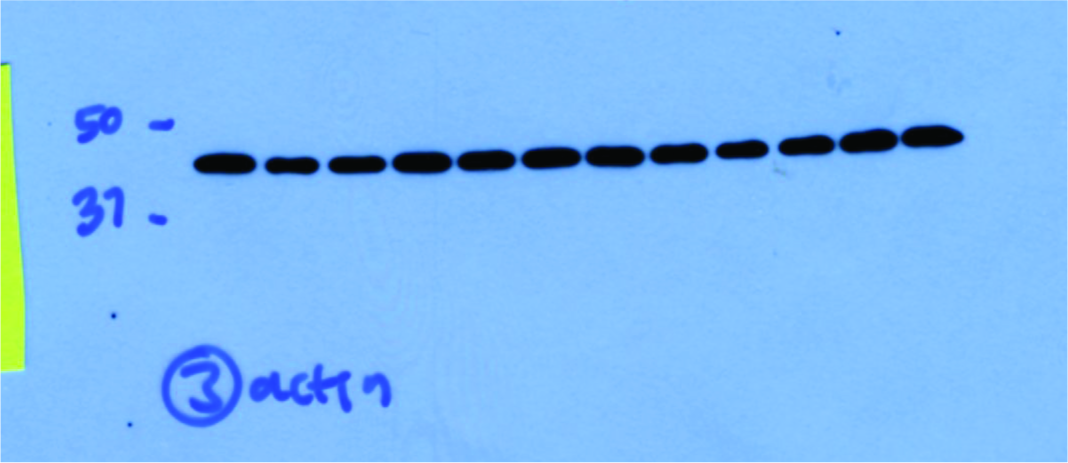

Supplement: Figure 2—figure supplement 3—source data 1. [file elife-83534-fig2-figsupp3-data1.zip › Figure 2-figure supplement 3-source data 1/Figure 2 figure supplement 3 Actin ACAT1 KO hACAT1(WT).tif]

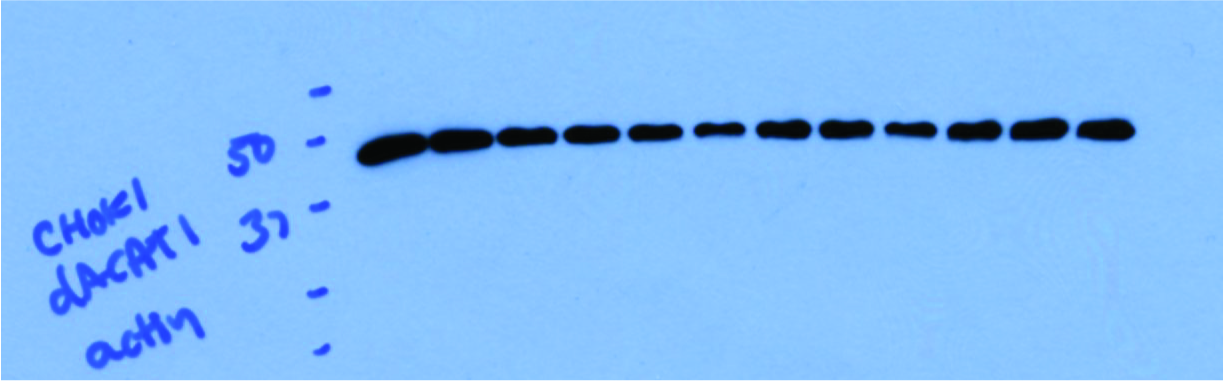

Supplement: Figure 2—figure supplement 3—source data 1. [file elife-83534-fig2-figsupp3-data1.zip › Figure 2-figure supplement 3-source data 1/Figure 2 figure supplement 3 Actin ACAT1 KO.tif]

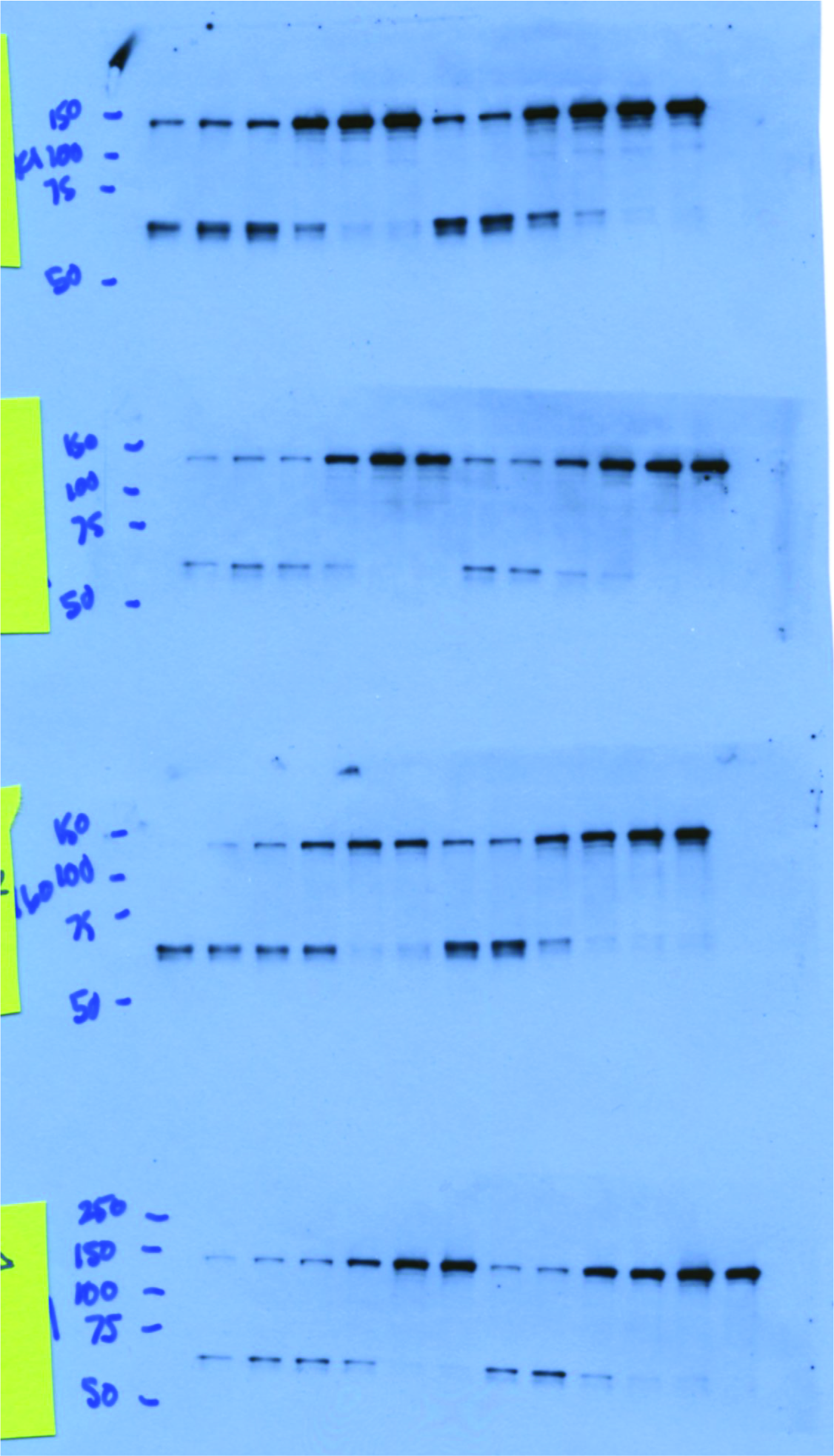

Supplement: Figure 2—figure supplement 3—source data 1. [file elife-83534-fig2-figsupp3-data1.zip › Figure 2-figure supplement 3-source data 1/Figure 2 figure supplement 3 SREBP2 for all cell lines.tif]

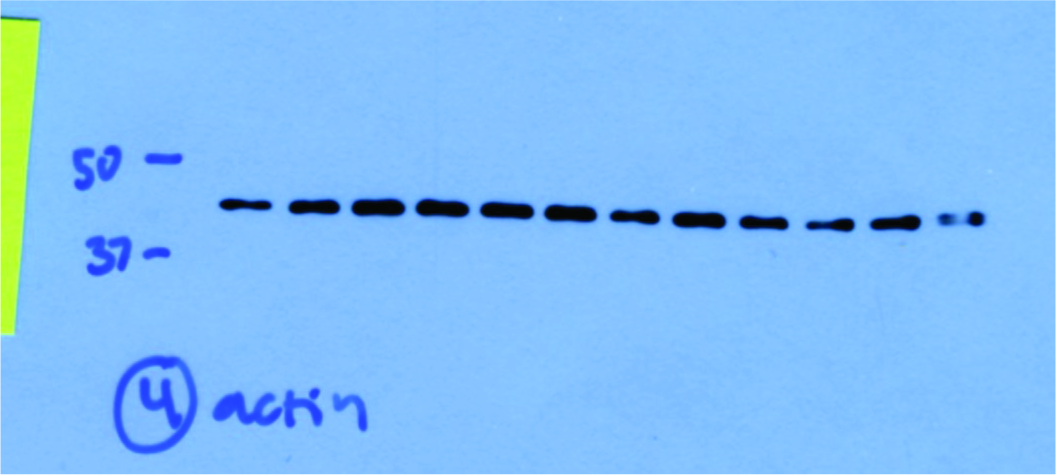

Supplement: Figure 2—figure supplement 3—source data 1. [file elife-83534-fig2-figsupp3-data1.zip › Figure 2-figure supplement 3-source data 1/Figure 2 figure supplement 3 Actin ACAT KO hACAT1(H460A).tif]

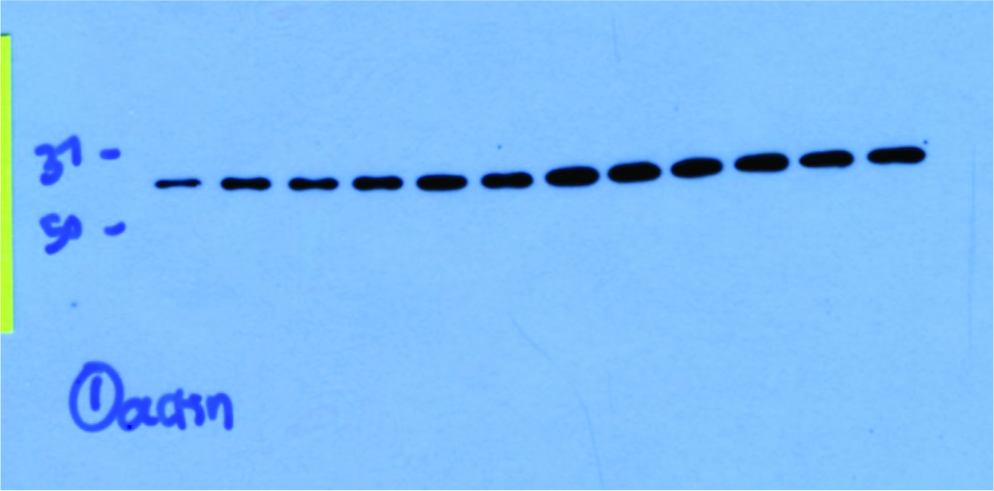

Supplement: Figure 2—figure supplement 3—source data 1. [file elife-83534-fig2-figsupp3-data1.zip › Figure 2-figure supplement 3-source data 1/Figure 2 figure supplement 3 Actin WT.tif]

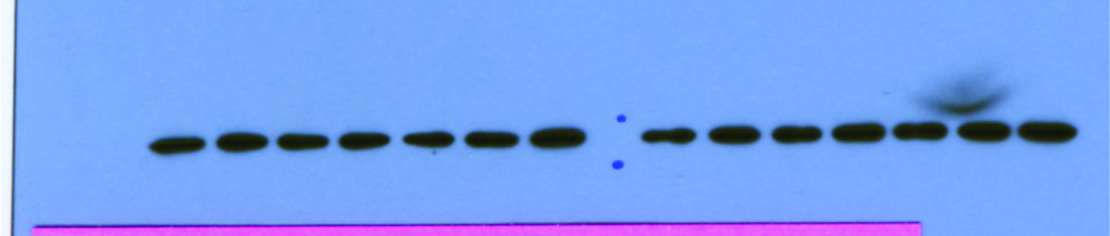

Supplement: Figure 2—figure supplement 4—source data 1. [file elife-83534-fig2-figsupp4-data1.zip › Figure 2-figure supplement 4-source data 1/Figure 2 figure supplement 4 Actin for WT cells.tif]

Figure 2 figure supplement 4 - Source Blots

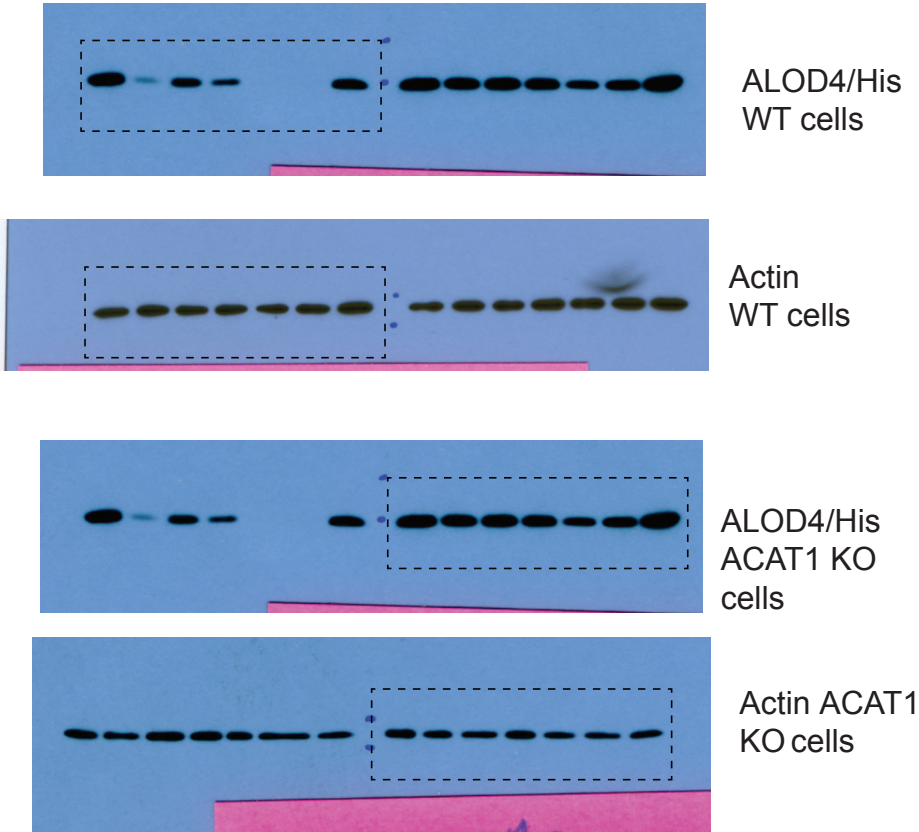

Supplement: Figure 2—figure supplement 4—source data 1. [file elife-83534-fig2-figsupp4-data1.zip › Figure 2-figure supplement 4-source data 1/Figure 2-figure supplement 4-souce data 1.pdf]

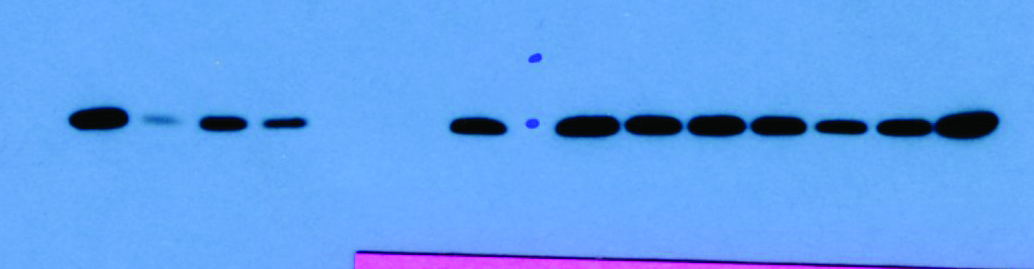

Supplement: Figure 2—figure supplement 4—source data 1. [file elife-83534-fig2-figsupp4-data1.zip › Figure 2-figure supplement 4-source data 1/Figure 2 figure supplement 4 ALOD4 for WT cells.tif]

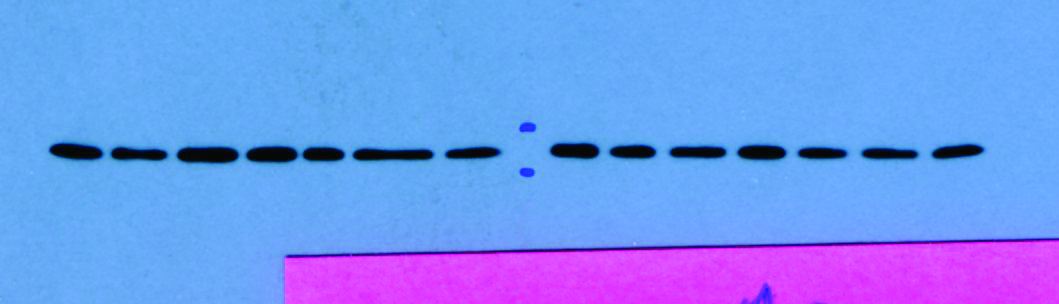

Supplement: Figure 2—figure supplement 4—source data 1. [file elife-83534-fig2-figsupp4-data1.zip › Figure 2-figure supplement 4-source data 1/Figure 2 figure supplement 4 Actin for ACAT1 KO cells.tif]

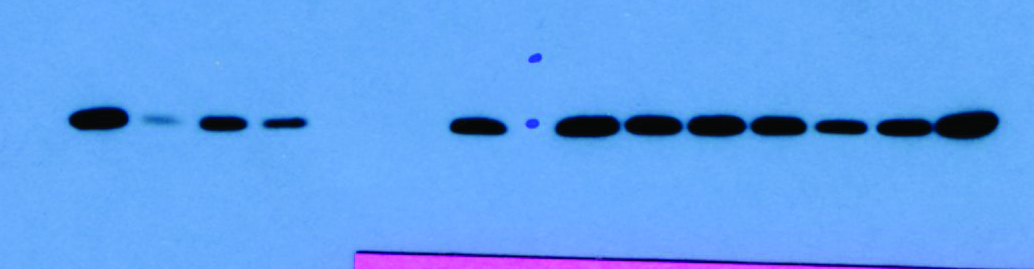

Supplement: Figure 2—figure supplement 4—source data 1. [file elife-83534-fig2-figsupp4-data1.zip › Figure 2-figure supplement 4-source data 1/Figure 2 figure supplement 4 ALOD4 for ACAT1 KO cells.tif]

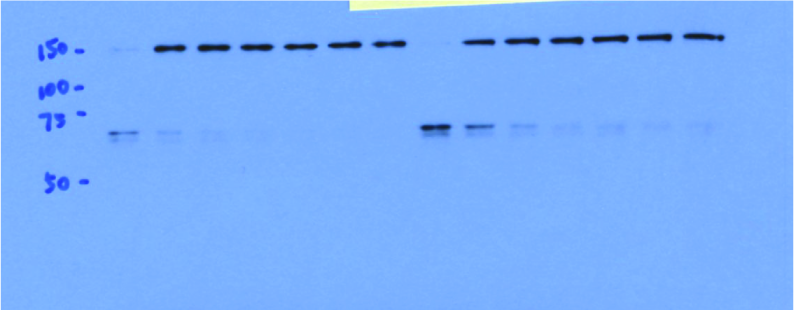

Supplement: Figure 3—source data 1. [file elife-83534-fig3-data1.zip › Figure 3-source data 1/Figure 3D SREBP2 for ACAT1 KO cells.tif]

# Figure 3 - Source Blots

C

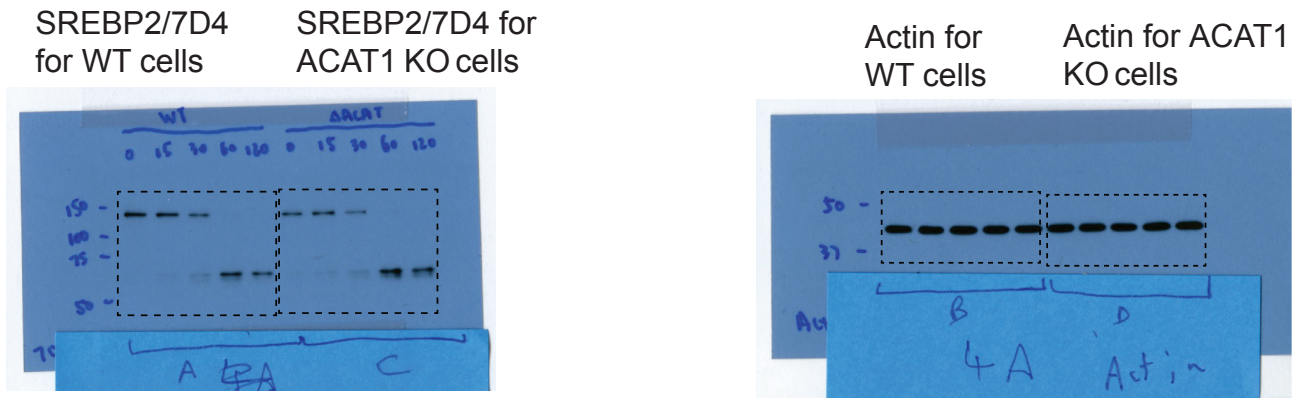

D

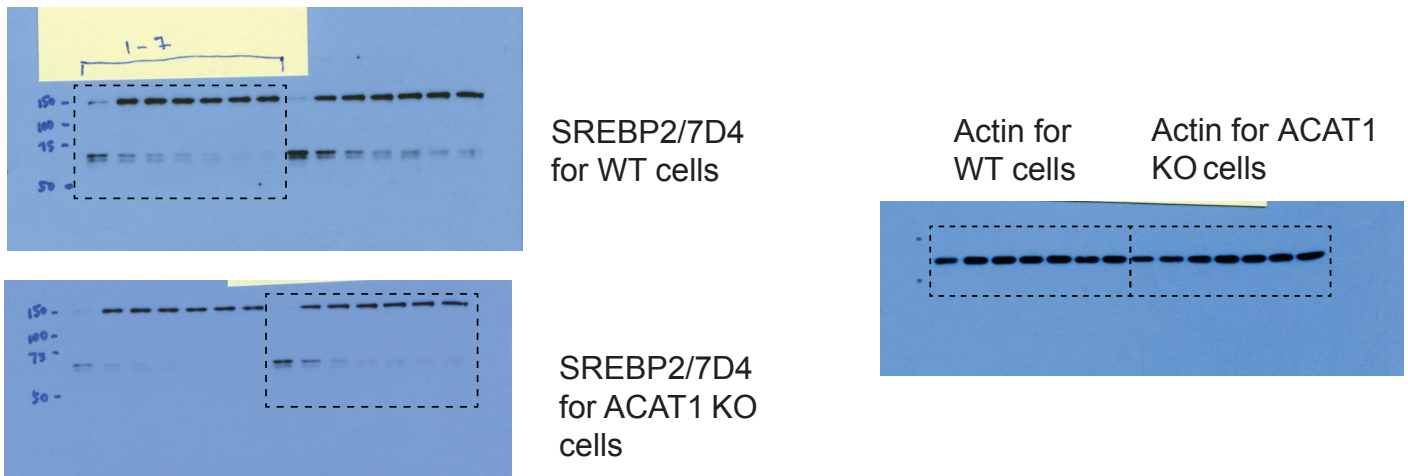

E

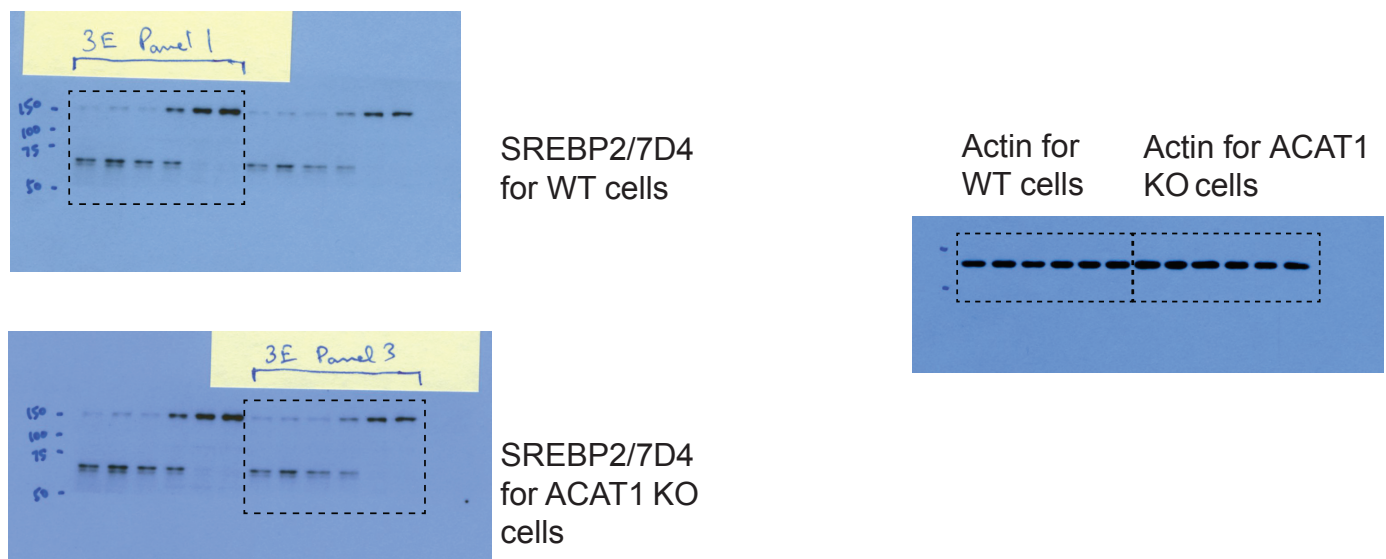

Supplement: Figure 3—source data 1. [file elife-83534-fig3-data1.zip › Figure 3-source data 1/Figure 3-soiuce data 1.pdf]

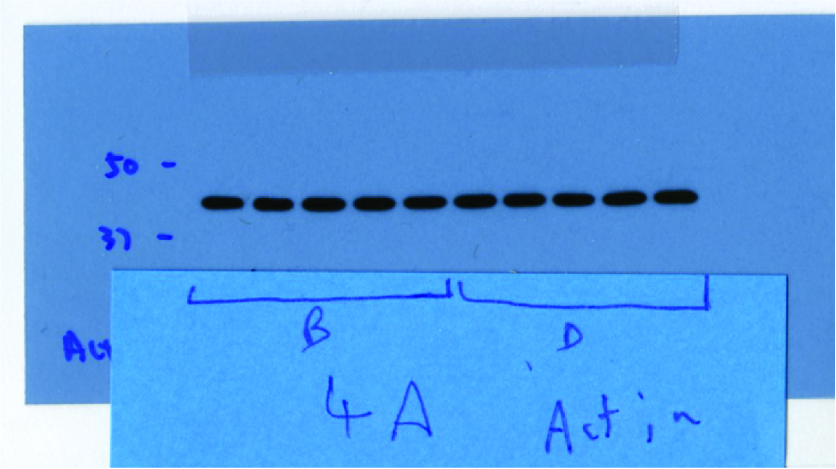

Supplement: Figure 3—source data 1. [file elife-83534-fig3-data1.zip › Figure 3-source data 1/Figure 3C Actin for WT and ACAT1 KO cells.tif]

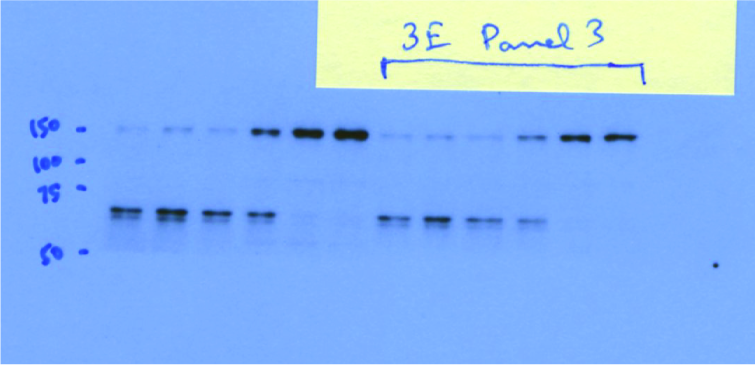

Supplement: Figure 3—source data 1. [file elife-83534-fig3-data1.zip › Figure 3-source data 1/Figure 3E SREBP2 for ACAT1 KO cells.tif]

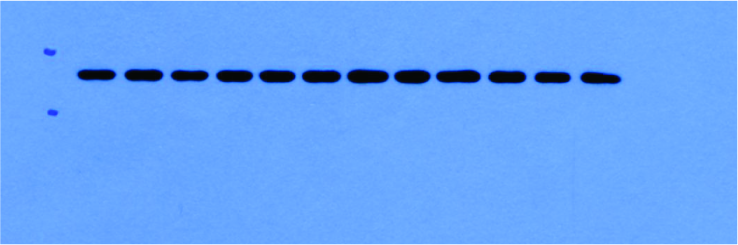

Supplement: Figure 3—source data 1. [file elife-83534-fig3-data1.zip › Figure 3-source data 1/Figure 3E Actin for WT and ACAT1 KO cells.tif]

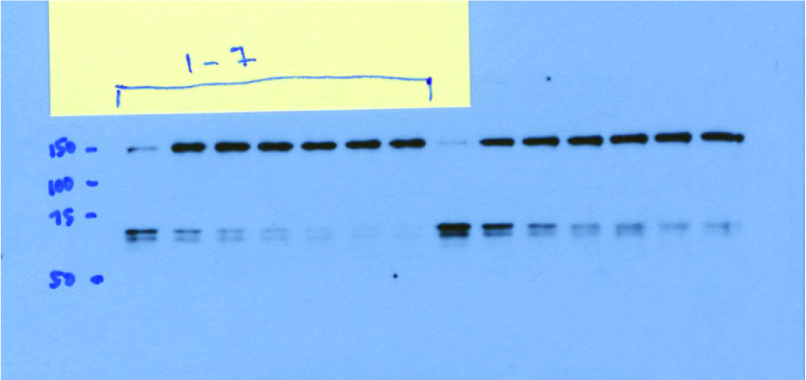

Supplement: Figure 3—source data 1. [file elife-83534-fig3-data1.zip › Figure 3-source data 1/Figure 3D SREBP2 for WT cells.tif]

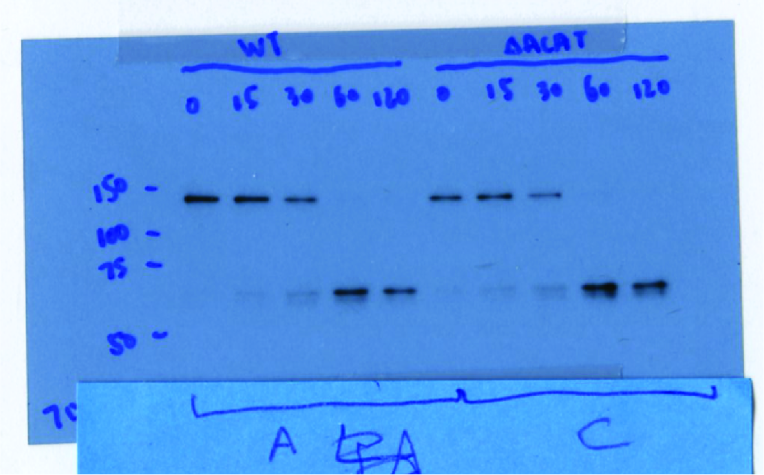

Supplement: Figure 3—source data 1. [file elife-83534-fig3-data1.zip › Figure 3-source data 1/Figure 3C SREBP2 for WT and ACAT1 KO cells.tif]

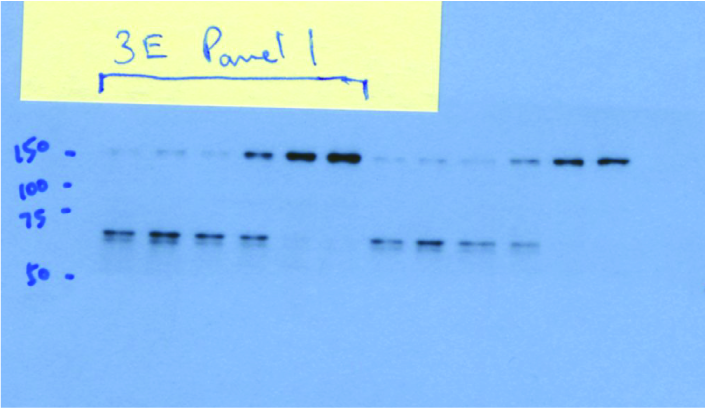

Supplement: Figure 3—source data 1. [file elife-83534-fig3-data1.zip › Figure 3-source data 1/Figure 3E SREBP2 for WT cells.tif]

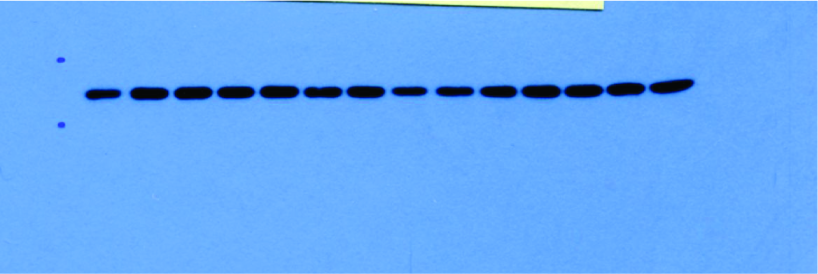

Supplement: Figure 3—source data 1. [file elife-83534-fig3-data1.zip › Figure 3-source data 1/Figure 3D Actin for WT and ACAT1 KO cells.tif]

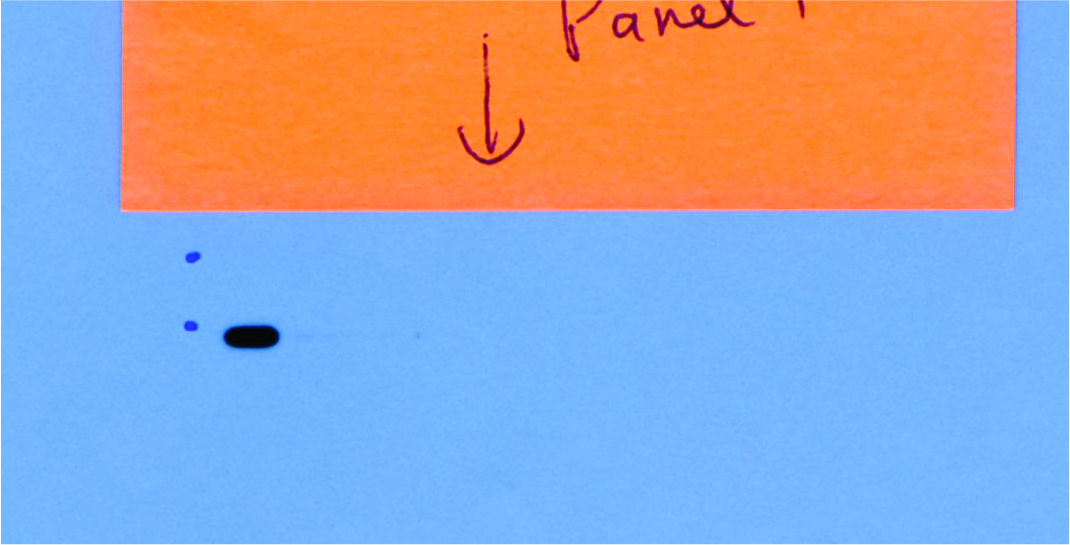

Supplement: Figure 4—source data 1. [file elife-83534-fig4-data1.zip › Figure 4-source data 1/Figure 4C ALOD4 for None-2.tif]

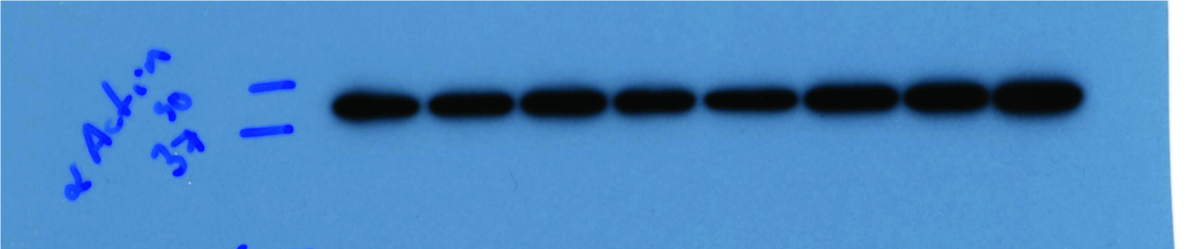

Supplement: Figure 4—source data 1. [file elife-83534-fig4-data1.zip › Figure 4-source data 1/Figure 4A Actin.tif]

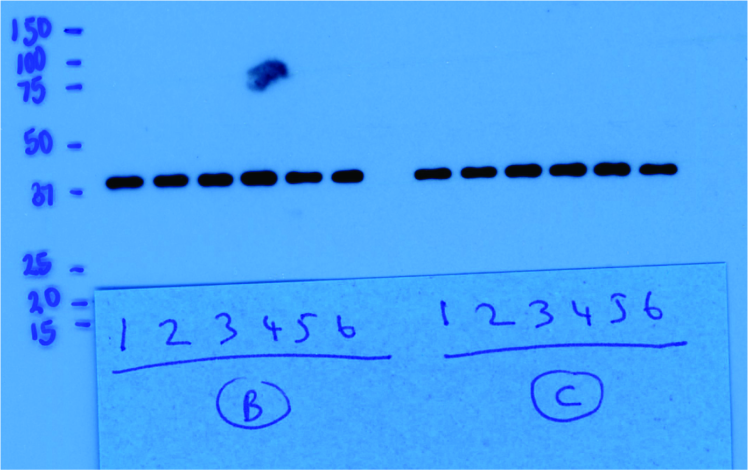

Supplement: Figure 4—source data 1. [file elife-83534-fig4-data1.zip › Figure 4-source data 1/Figure 4B BP1c Actin.tif]

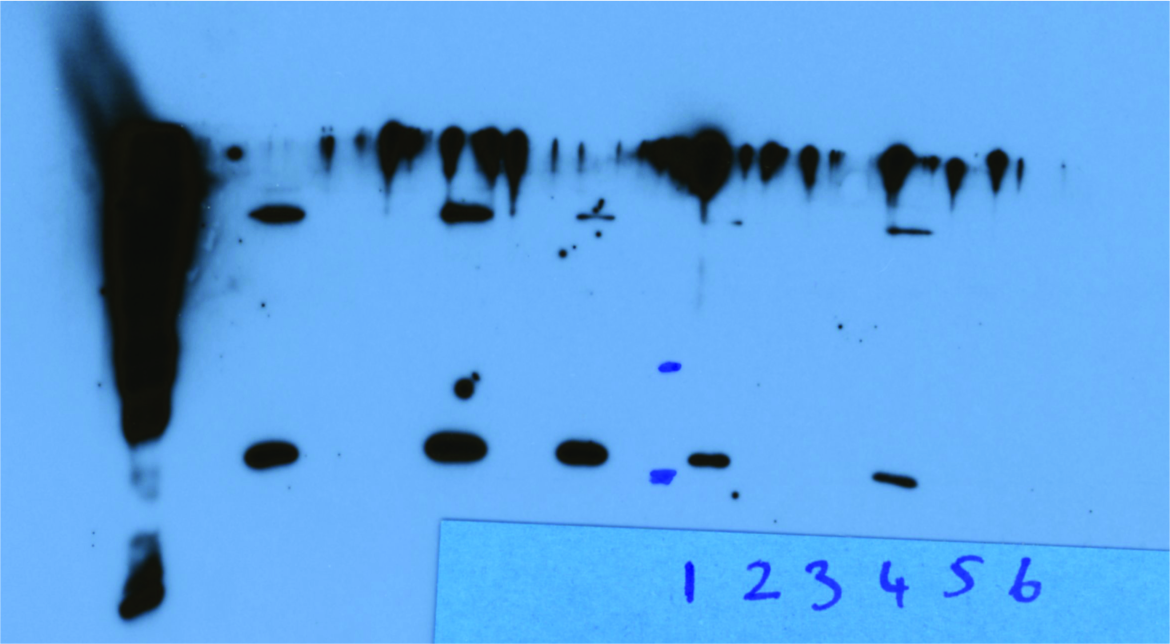

Supplement: Figure 4—source data 1. [file elife-83534-fig4-data1.zip › Figure 4-source data 1/Figure 4B BP1c ALOD4.tif]

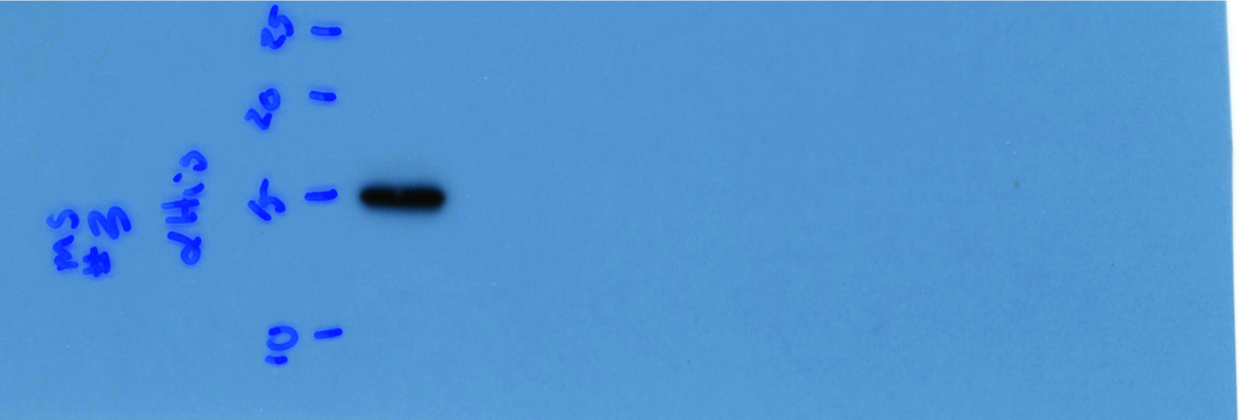

Supplement: Figure 4—source data 1. [file elife-83534-fig4-data1.zip › Figure 4-source data 1/Figure 4A ALOD4.tif]

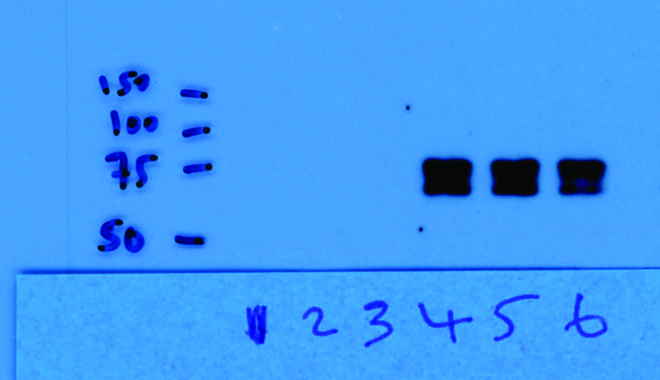

Supplement: Figure 4—source data 1. [file elife-83534-fig4-data1.zip › Figure 4-source data 1/Figure 4B BP1a FLAG.tif]

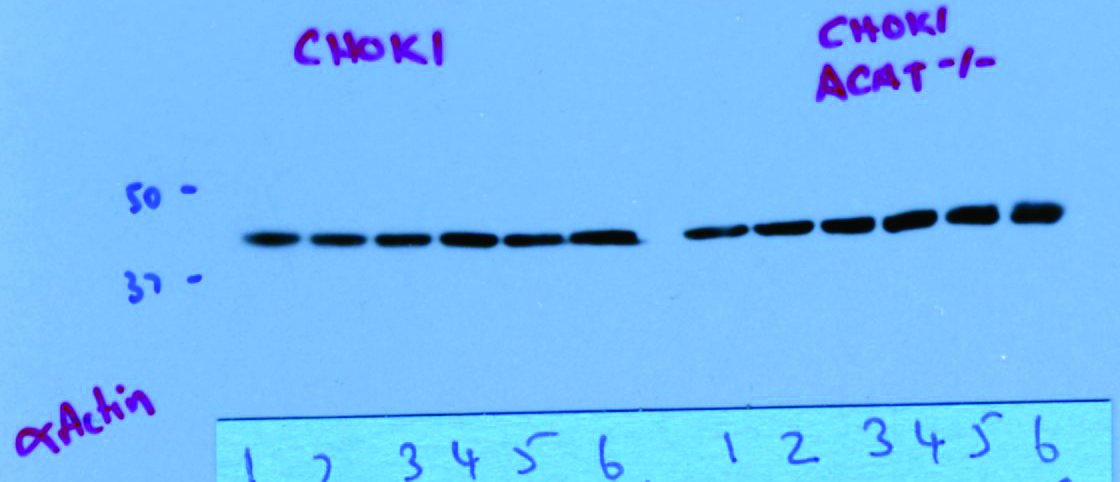

Supplement: Figure 4—source data 1. [file elife-83534-fig4-data1.zip › Figure 4-source data 1/Figure 4D Actin for WT cells.tif]

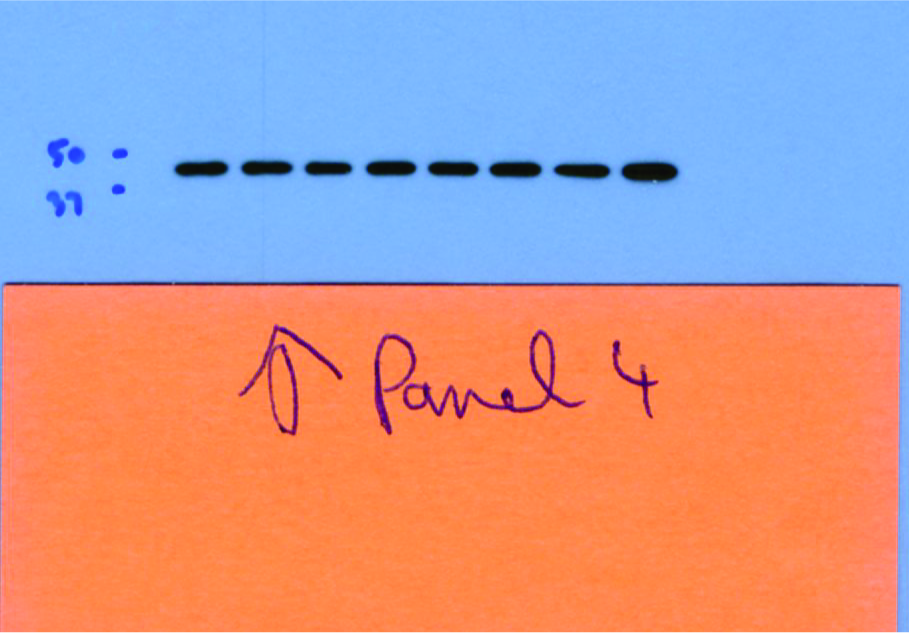

Supplement: Figure 4—source data 1. [file elife-83534-fig4-data1.zip › Figure 4-source data 1/Figure 4C Actin for Inhibitor.tif]

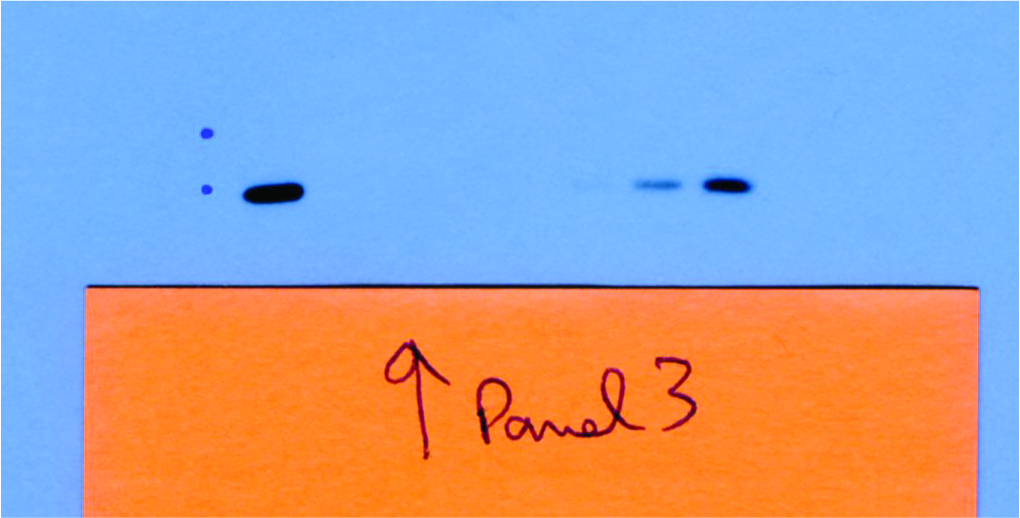

Supplement: Figure 4—source data 1. [file elife-83534-fig4-data1.zip › Figure 4-source data 1/Figure 4C ALOD4 for Inhibitor.tif]

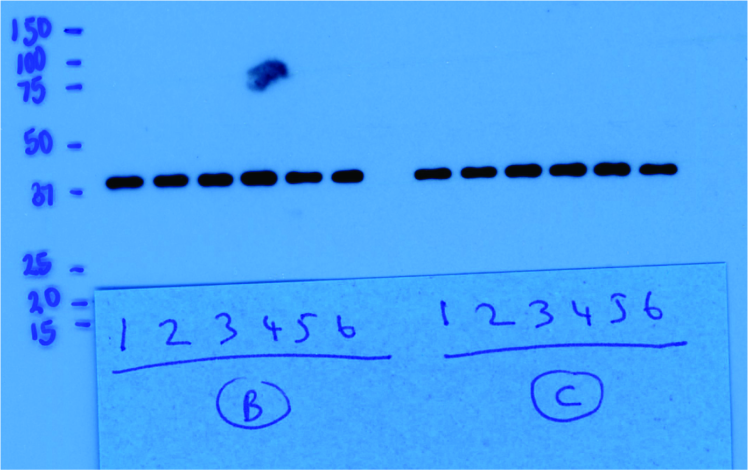

Supplement: Figure 4—source data 1. [file elife-83534-fig4-data1.zip › Figure 4-source data 1/Figure 4B BP2 Actin.tif]

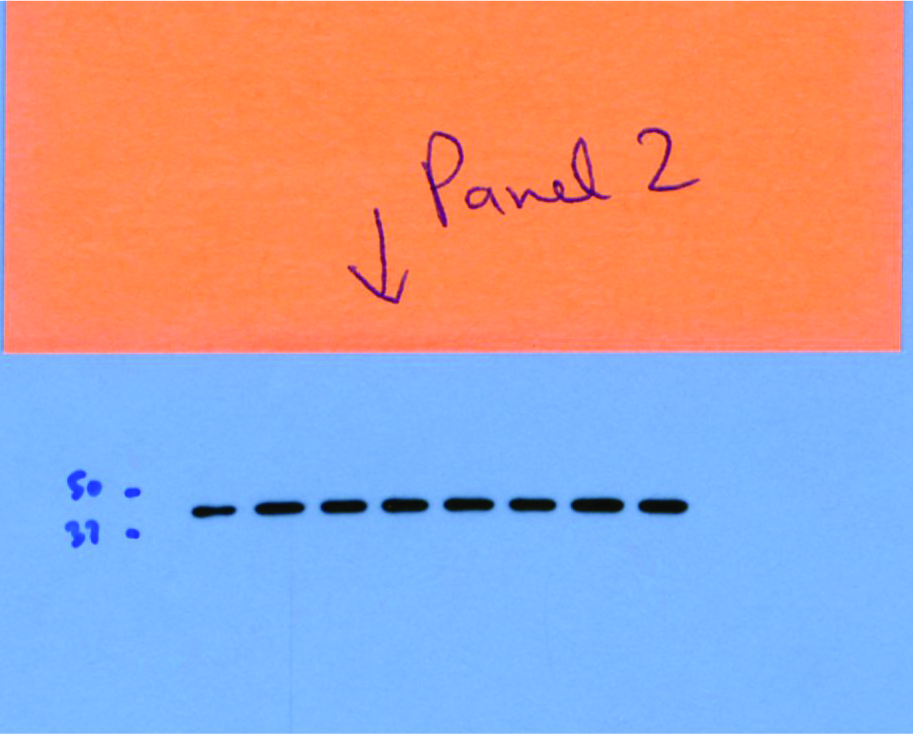

Supplement: Figure 4—source data 1. [file elife-83534-fig4-data1.zip › Figure 4-source data 1/Figure 4C Actin for None-2.tif]

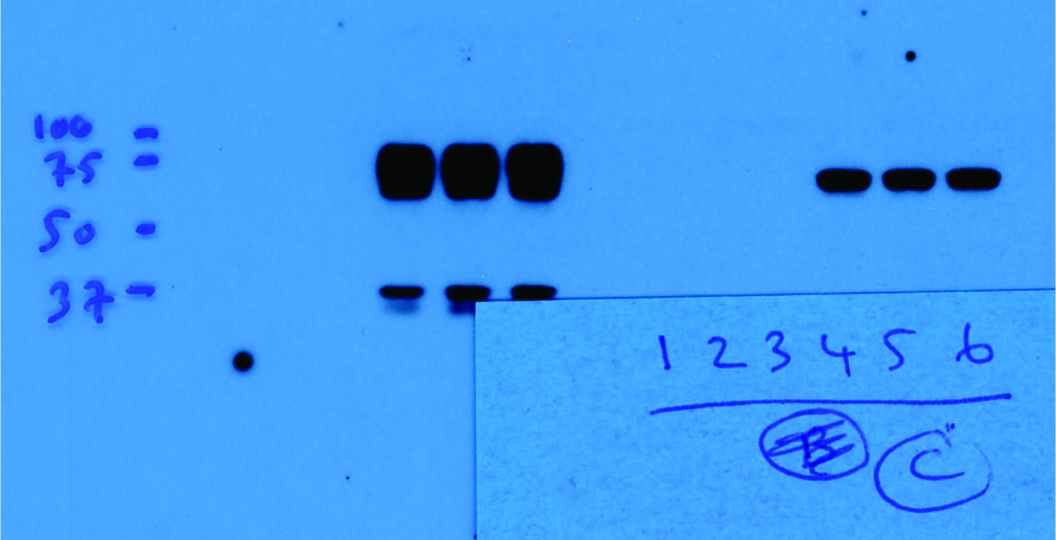

Supplement: Figure 4—source data 1. [file elife-83534-fig4-data1.zip › Figure 4-source data 1/Figure 4B BP2 FLAG.tif]

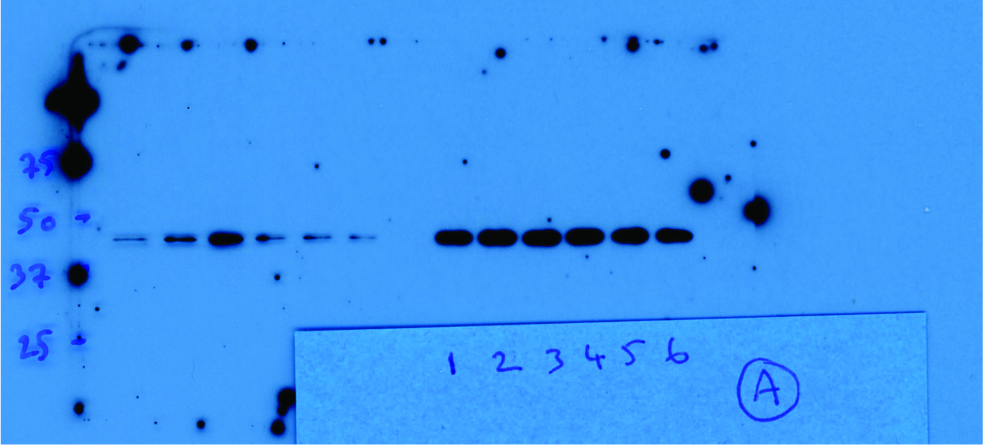

Supplement: Figure 4—source data 1. [file elife-83534-fig4-data1.zip › Figure 4-source data 1/Figure 4B BP1a Actin.tif]

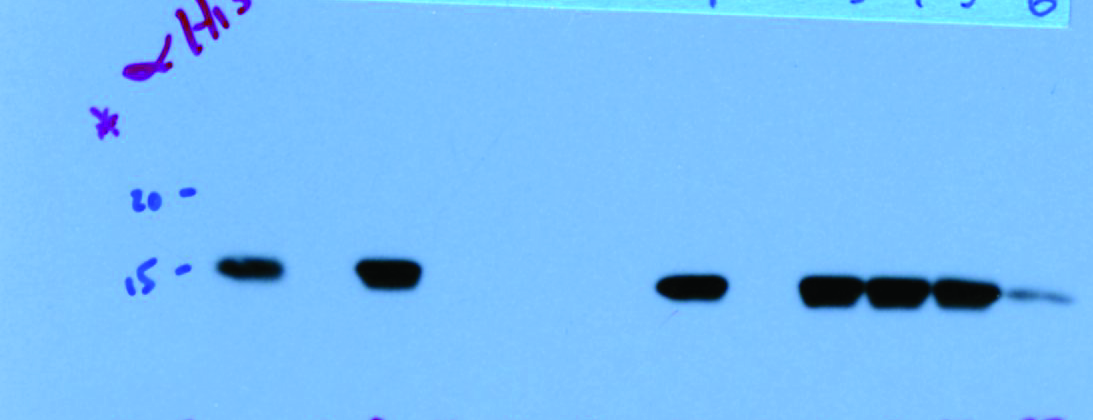

Supplement: Figure 4—source data 1. [file elife-83534-fig4-data1.zip › Figure 4-source data 1/Figure 4D ALOD4 for WT cells.tif]

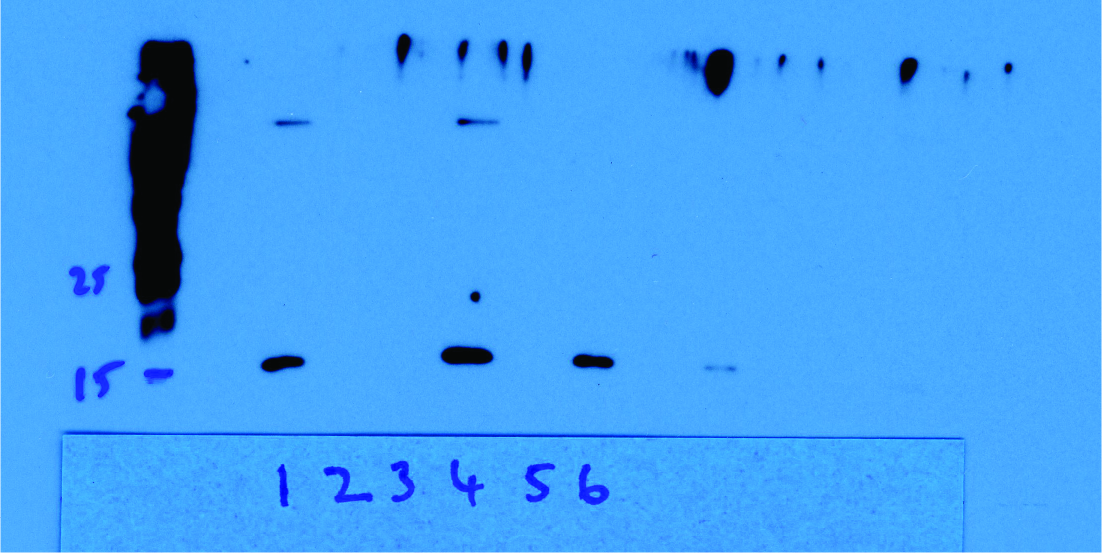

Supplement: Figure 4—source data 1. [file elife-83534-fig4-data1.zip › Figure 4-source data 1/Figure 4B BP1a ALOD4.tif]

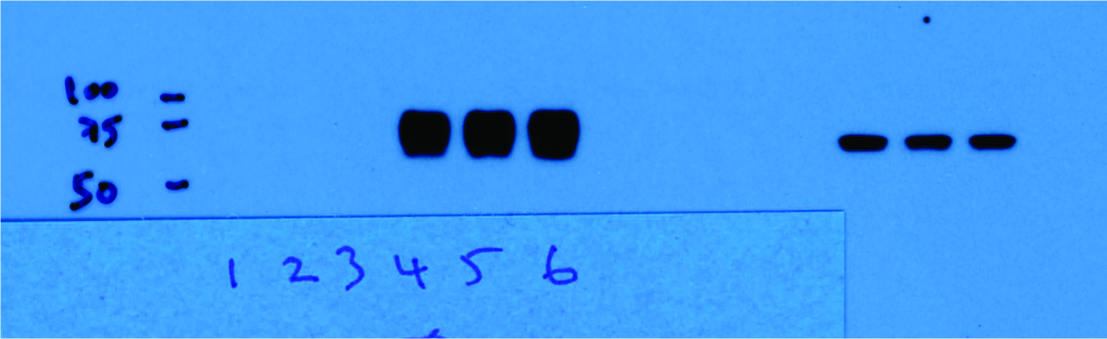

Supplement: Figure 4—source data 1. [file elife-83534-fig4-data1.zip › Figure 4-source data 1/Figure 4B BP1c FLAG.tif]

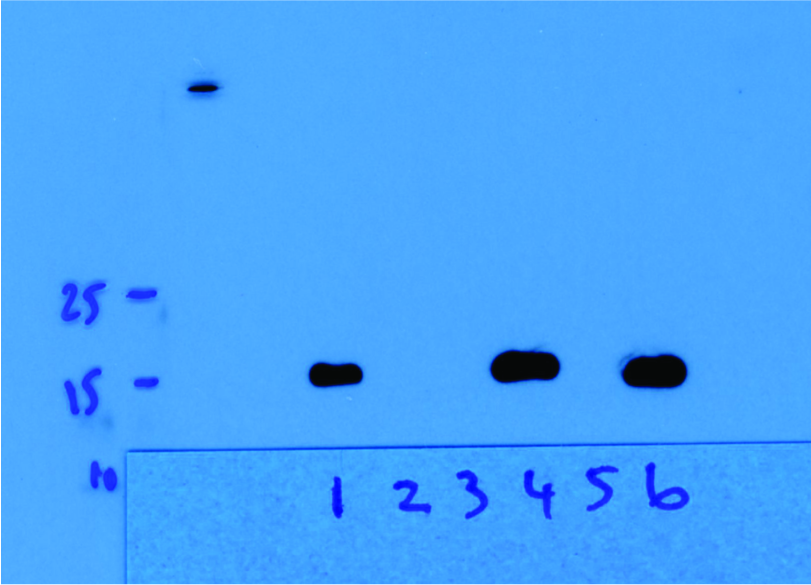

Supplement: Figure 4—source data 1. [file elife-83534-fig4-data1.zip › Figure 4-source data 1/Figure 4B BP2 ALOD4.tif]

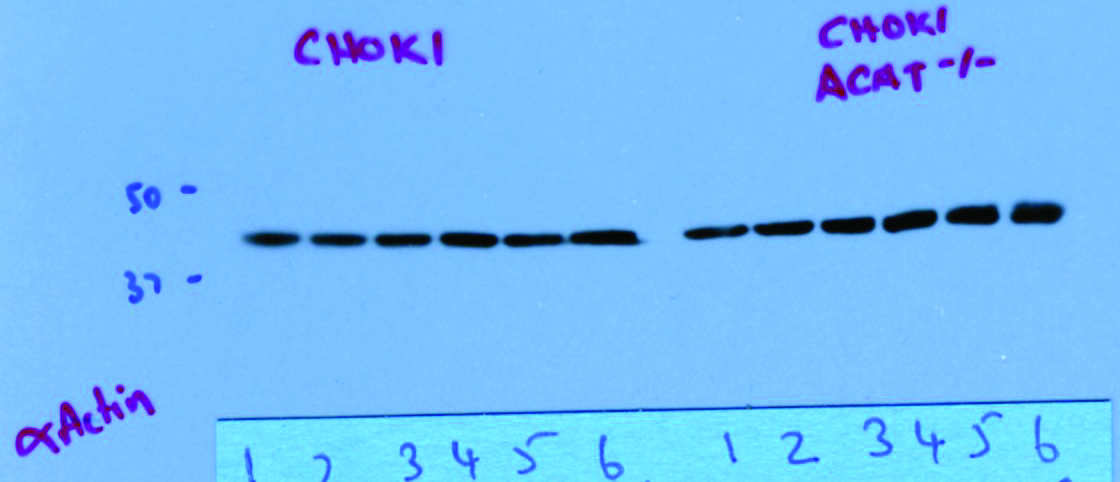

Supplement: Figure 4—source data 1. [file elife-83534-fig4-data1.zip › Figure 4-source data 1/Figure 4D Actin for ACAT1 KO cells.tif]

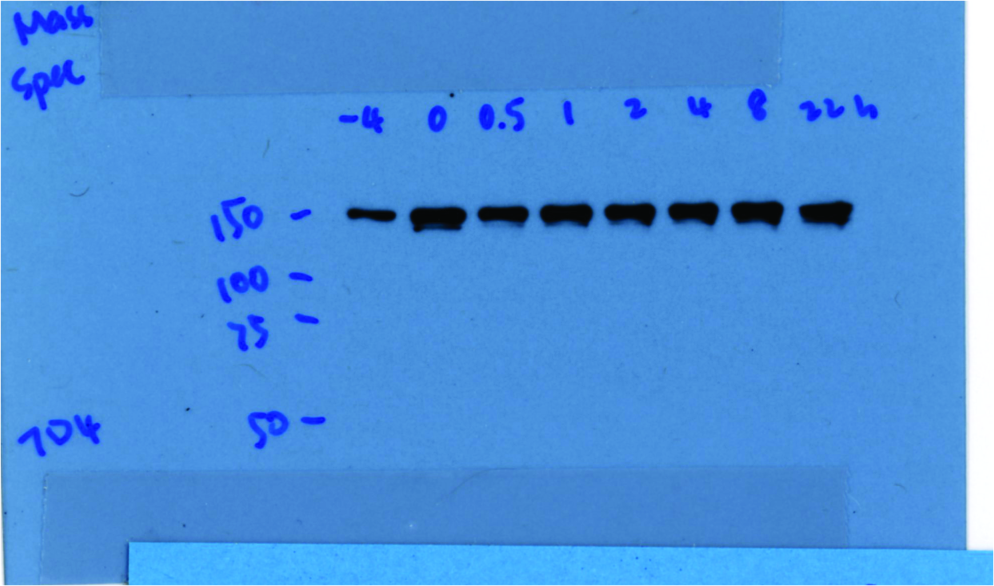

Supplement: Figure 4—source data 1. [file elife-83534-fig4-data1.zip › Figure 4-source data 1/Figure 4A SREBP2.tif]

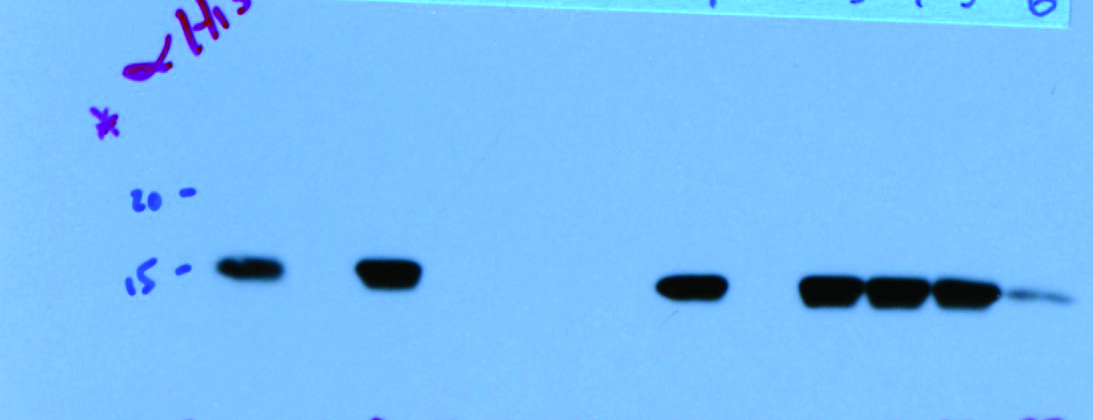

Supplement: Figure 4—source data 1. [file elife-83534-fig4-data1.zip › Figure 4-source data 1/Figure 4D ALOD4 for ACAT1 KO cells.tif]

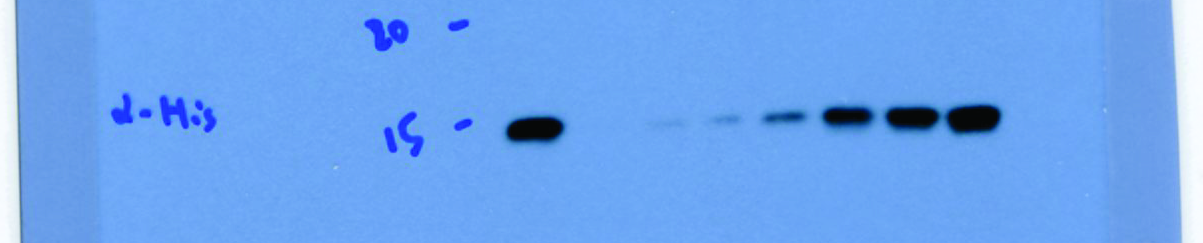

Supplement: Figure 4—figure supplement 1—source data 1. [file elife-83534-fig4-figsupp1-data1.zip › Figure 4-figure supplement 1-source data 1/Figure 4 supplement 1 ALOD4.tif]

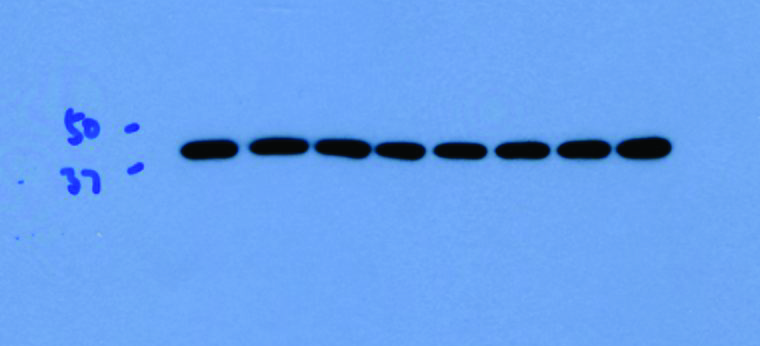

Supplement: Figure 4—figure supplement 1—source data 1. [file elife-83534-fig4-figsupp1-data1.zip › Figure 4-figure supplement 1-source data 1/Figure 4 supplement 1 Actin.tif]

## Figure 4 figure supplement 1- Source Blots

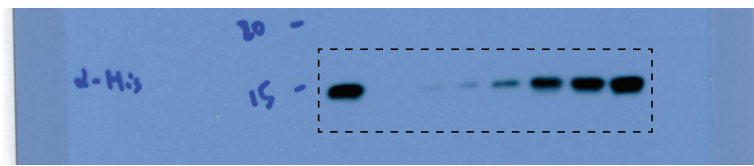

ALOD4/His

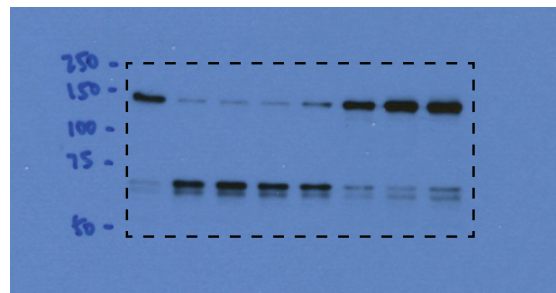

SREBP2/7D4

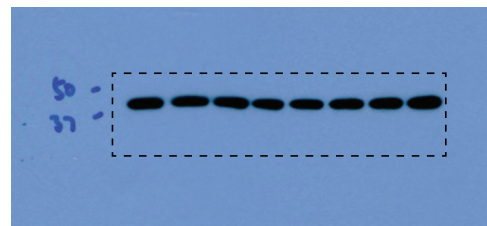

Actin

Supplement: Figure 4—figure supplement 1—source data 1. [file elife-83534-fig4-figsupp1-data1.zip › Figure 4-figure supplement 1-source data 1/Figure 4-figure 4 supplement 1-source data 1.pdf]

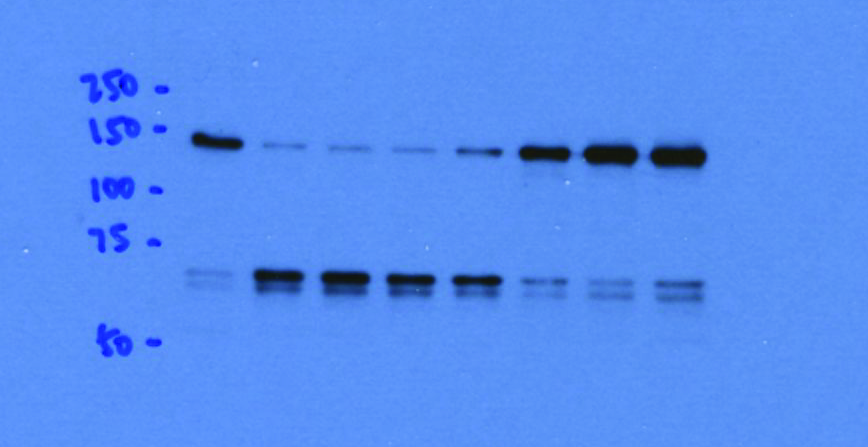

Supplement: Figure 4—figure supplement 1—source data 1. [file elife-83534-fig4-figsupp1-data1.zip › Figure 4-figure supplement 1-source data 1/Figure 4 supplement 1 SREBP2.tif]

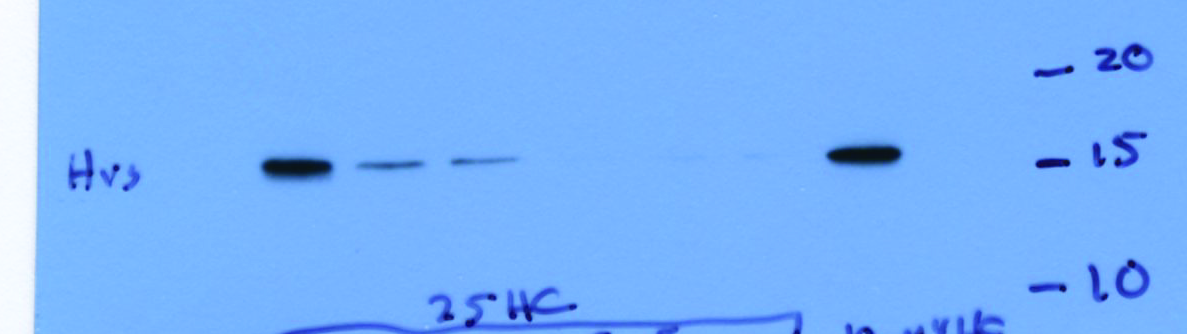

Supplement: Figure 7—figure supplement 1—source data 1. [file elife-83534-fig7-figsupp1-data1.zip › Figure 7-figure supplement 1-source data 1/Figure 7 figure supplement 1C ALOD4 WT.tif]

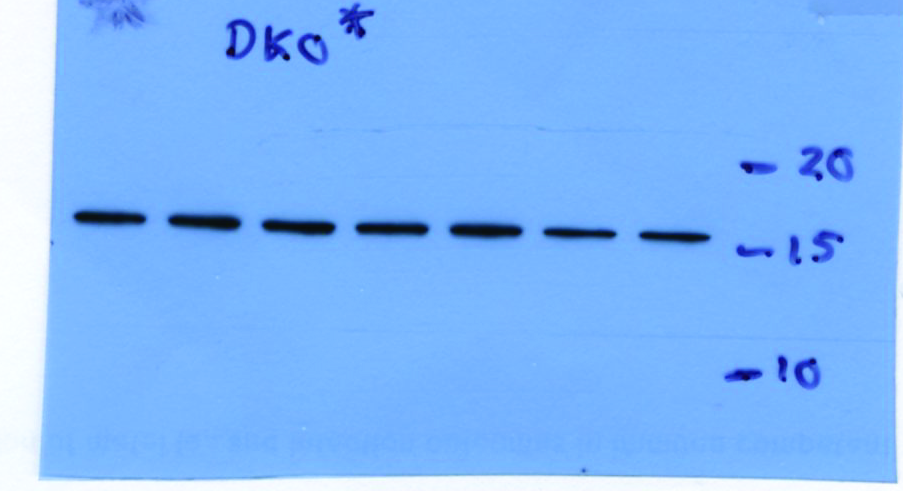

Supplement: Figure 7—figure supplement 1—source data 1. [file elife-83534-fig7-figsupp1-data1.zip › Figure 7-figure supplement 1-source data 1/Figure 7 figure supplement 1C ALOD4 ACAT-null.tif]

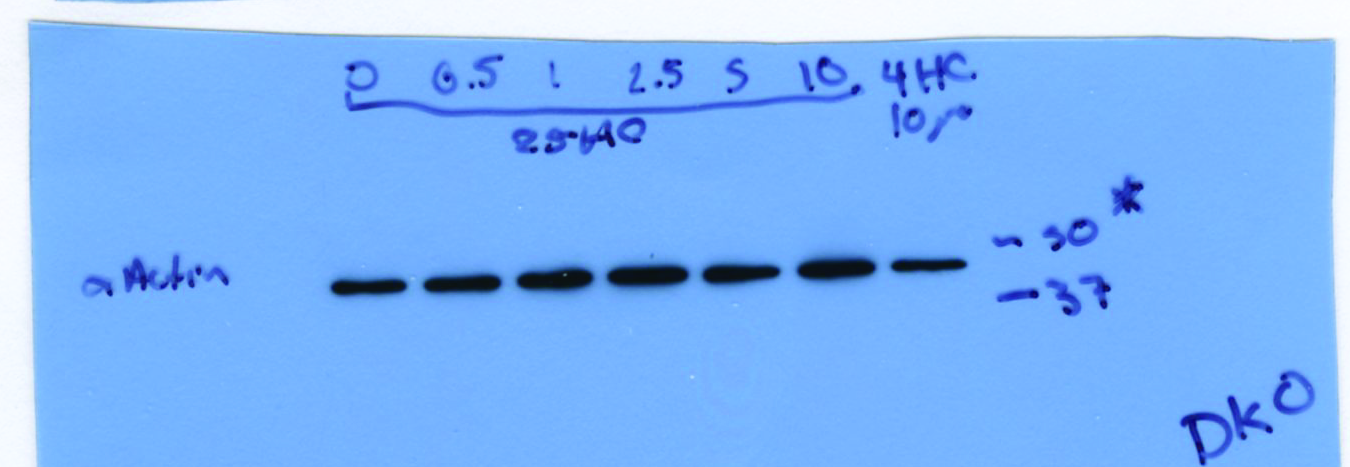

Supplement: Figure 7—figure supplement 1—source data 1. [file elife-83534-fig7-figsupp1-data1.zip › Figure 7-figure supplement 1-source data 1/Figure 7 figure supplement 1C Actin for ACAT-null.tif]

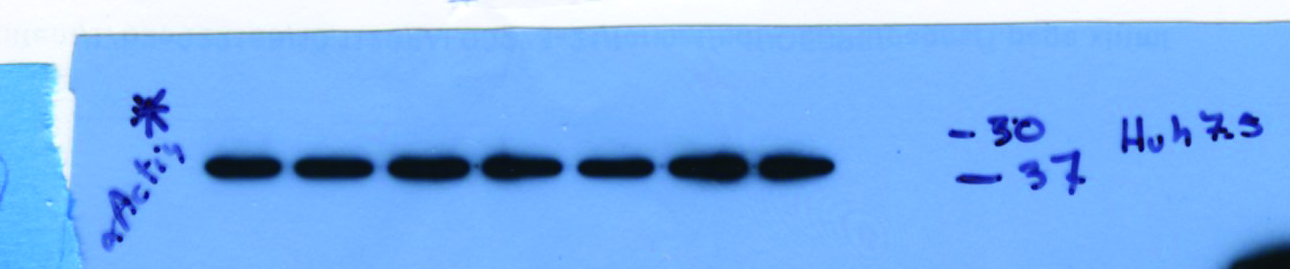

Supplement: Figure 7—figure supplement 1—source data 1. [file elife-83534-fig7-figsupp1-data1.zip › Figure 7-figure supplement 1-source data 1/Figure 7 figure supplement 1C Actin for WT.tif]
