# Supplementary material for: A concerted mechanism involving ACAT and SREBPs by which oxysterols deplete accessible cholesterol to restrict microbial infection
Source: eLife. 2023 Jan 25;12:e83534. doi: 10.7554/eLife.83534 (PMC9925056; doi:10.7554/eLife.83534)
Supplement: Figure 2—source data 1. [file elife-83534-fig2-data1.zip › Figure 2-source data 1/Figure 2-source data 1.pdf]

Figure 2 - Source Blots

A

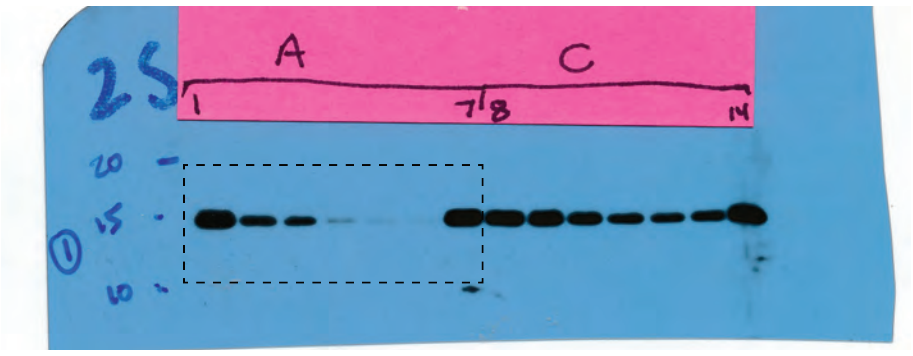

ALOD4/His for WT cells

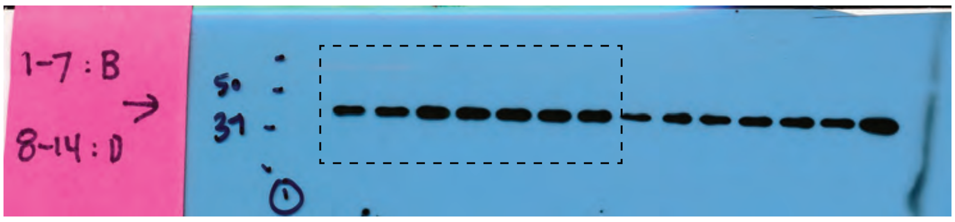

Actin for WT cells

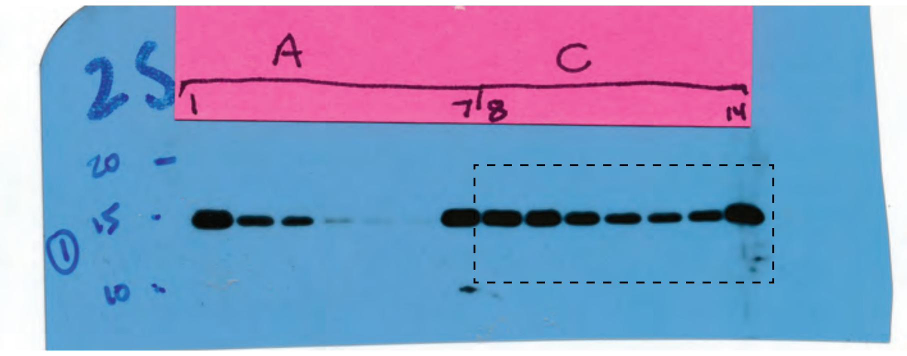

ALOD4/His for ACAT1 KO cells

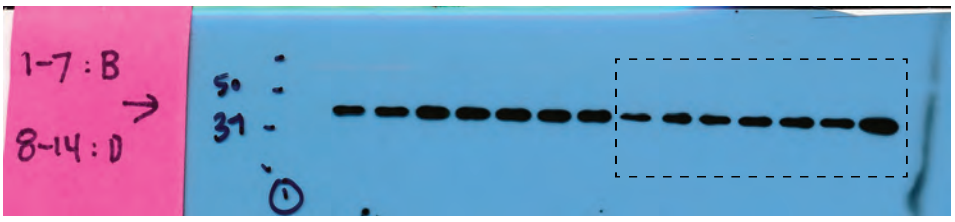

Actin for ACAT1 KO cells

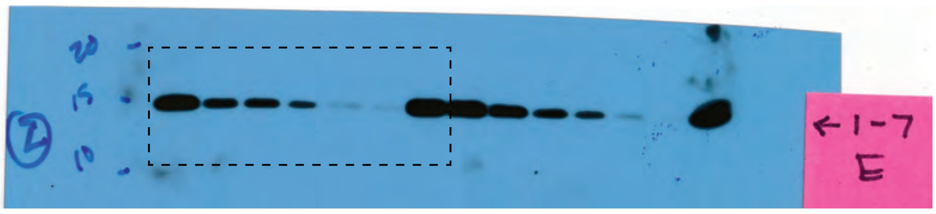

ALOD4/His
for ACAT1 KO;hACAT1(WT)
cells

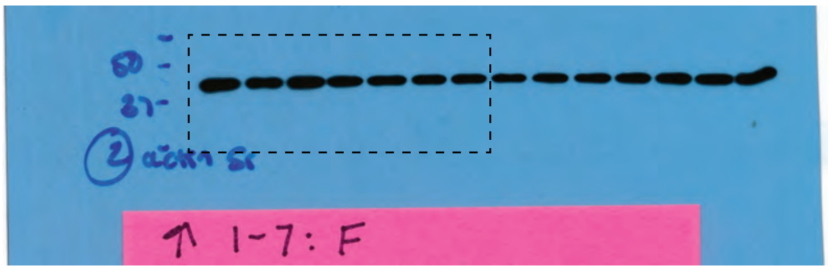

Actin
for ACAT1 KO;hACAT1(WT)
cells

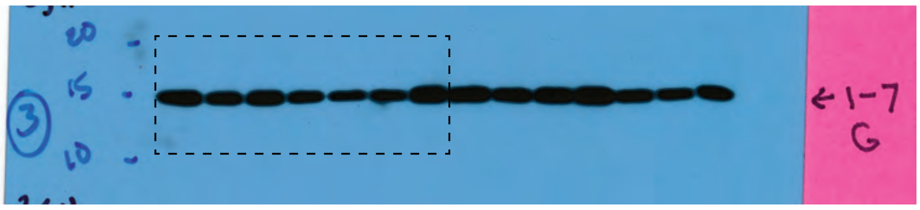

ALOD4/His
for ACAT1 KO;hACAT1(H460A)
cells

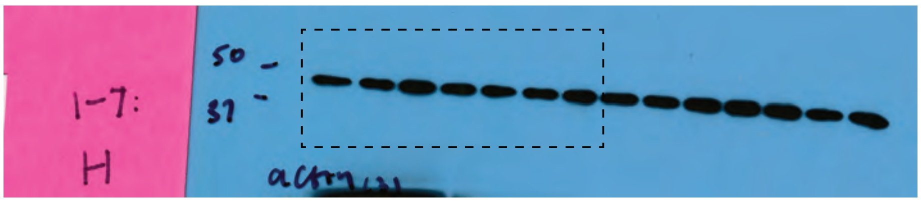

Actin
for ACAT1 KO;hACAT1(H460A)
cells

C

ALOD4/His
for WT cells

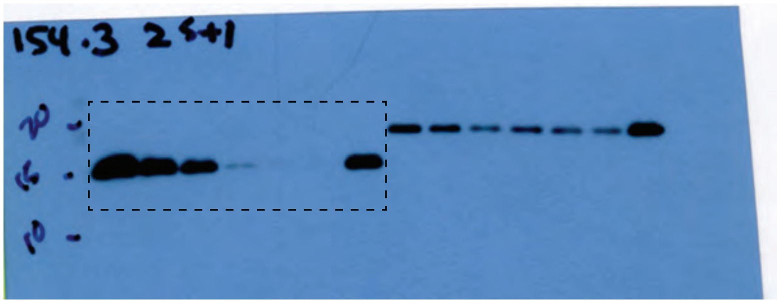

Actin
for WT cells (ALOD4)

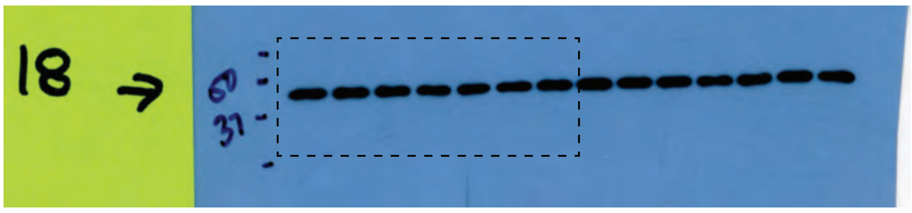

ALOD4/His for
SCAP-deficient
cells

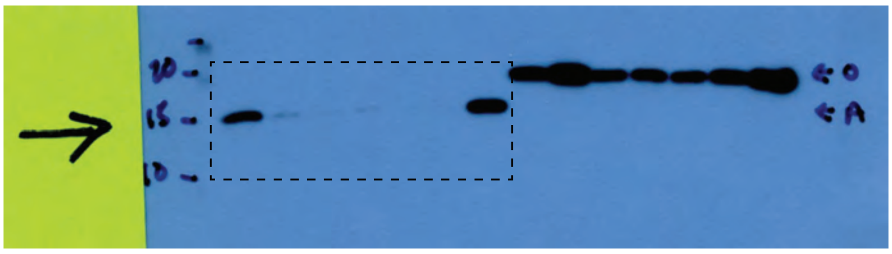

Actin
for SCAP-deficient cells
(ALOD4)

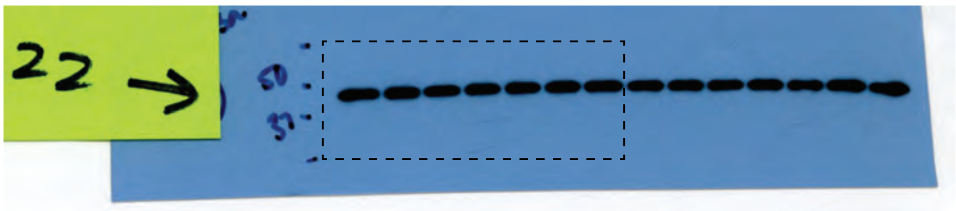

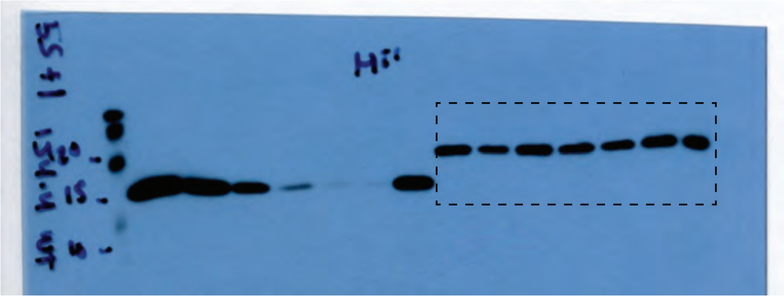

OlyA/His
for WT cells

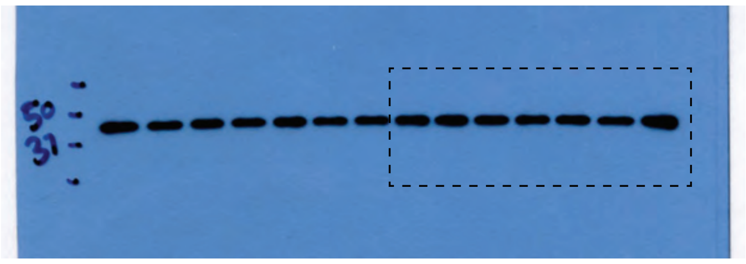

Actin
for WT cells (OlyA)

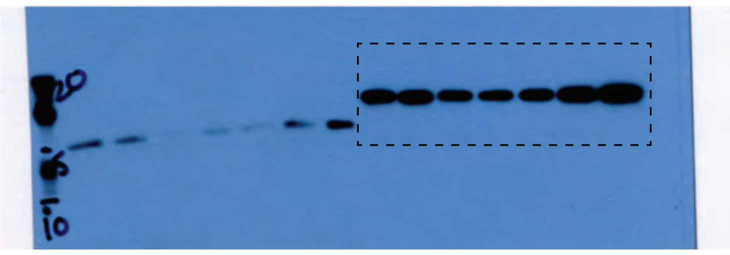

OlyA/His
for SCAP-
deficient cells

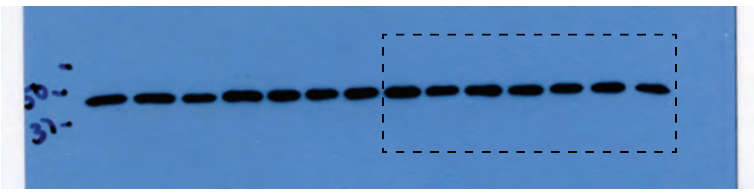

Actin
for SCAP-deficient
cells (OlyA)

D

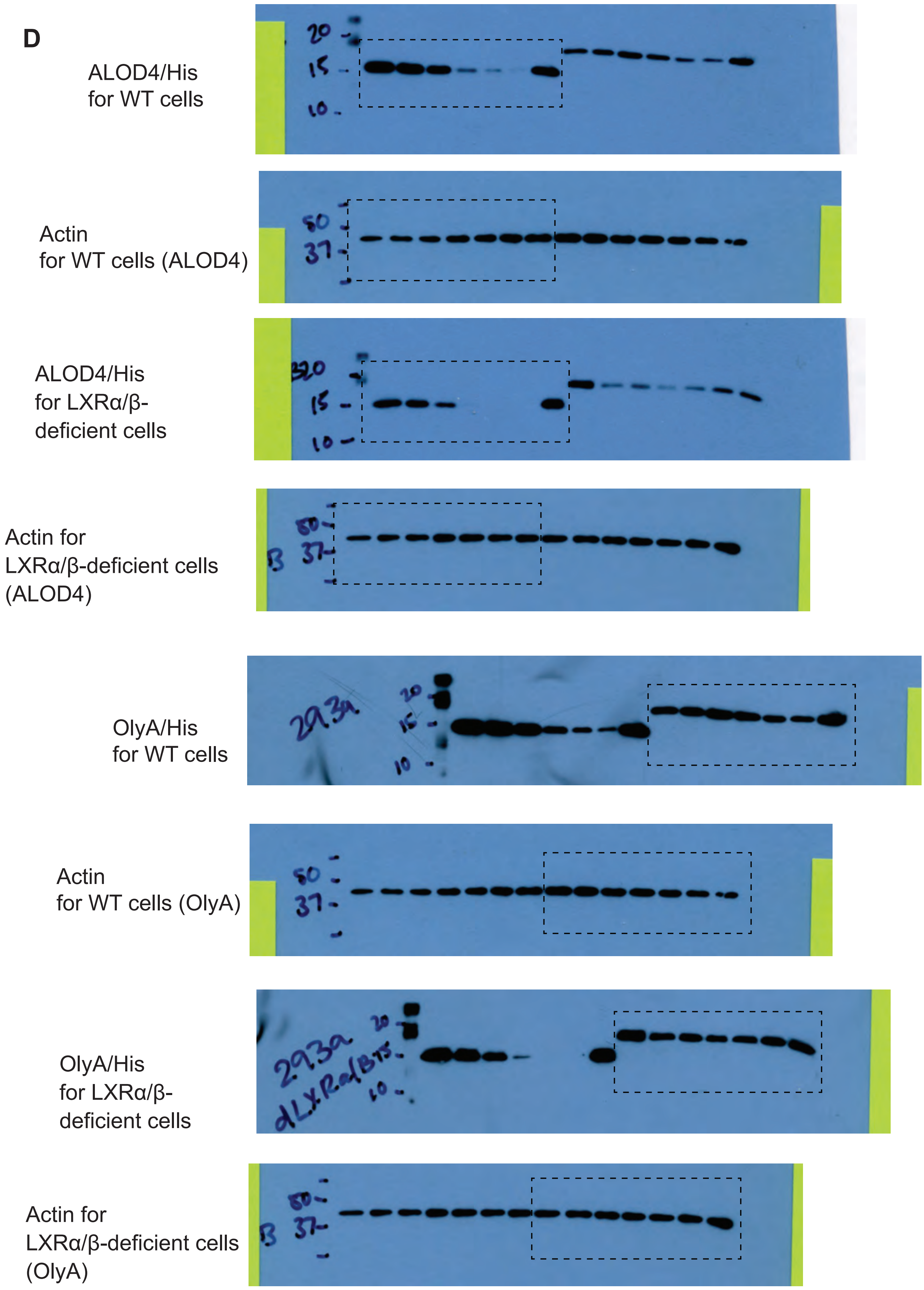
ALOD4/His for WT cells
Actin for WT cells (ALOD4)
ALOD4/His for LXRα/β-deficient cells
Actin for LXRα/β-deficient cells (ALOD4)
OlyA/His for WT cells
Actin for WT cells (OlyA)
OlyA/His for LXRα/β-deficient cells
Actin for LXRα/β-deficient cells (OlyA)
