# Supplementary material for: A concerted mechanism involving ACAT and SREBPs by which oxysterols deplete accessible cholesterol to restrict microbial infection
Source: eLife. 2023 Jan 25;12:e83534. doi: 10.7554/eLife.83534 (PMC9925056; doi:10.7554/eLife.83534)

Figure 2 figure supplement 3 - Source Blots

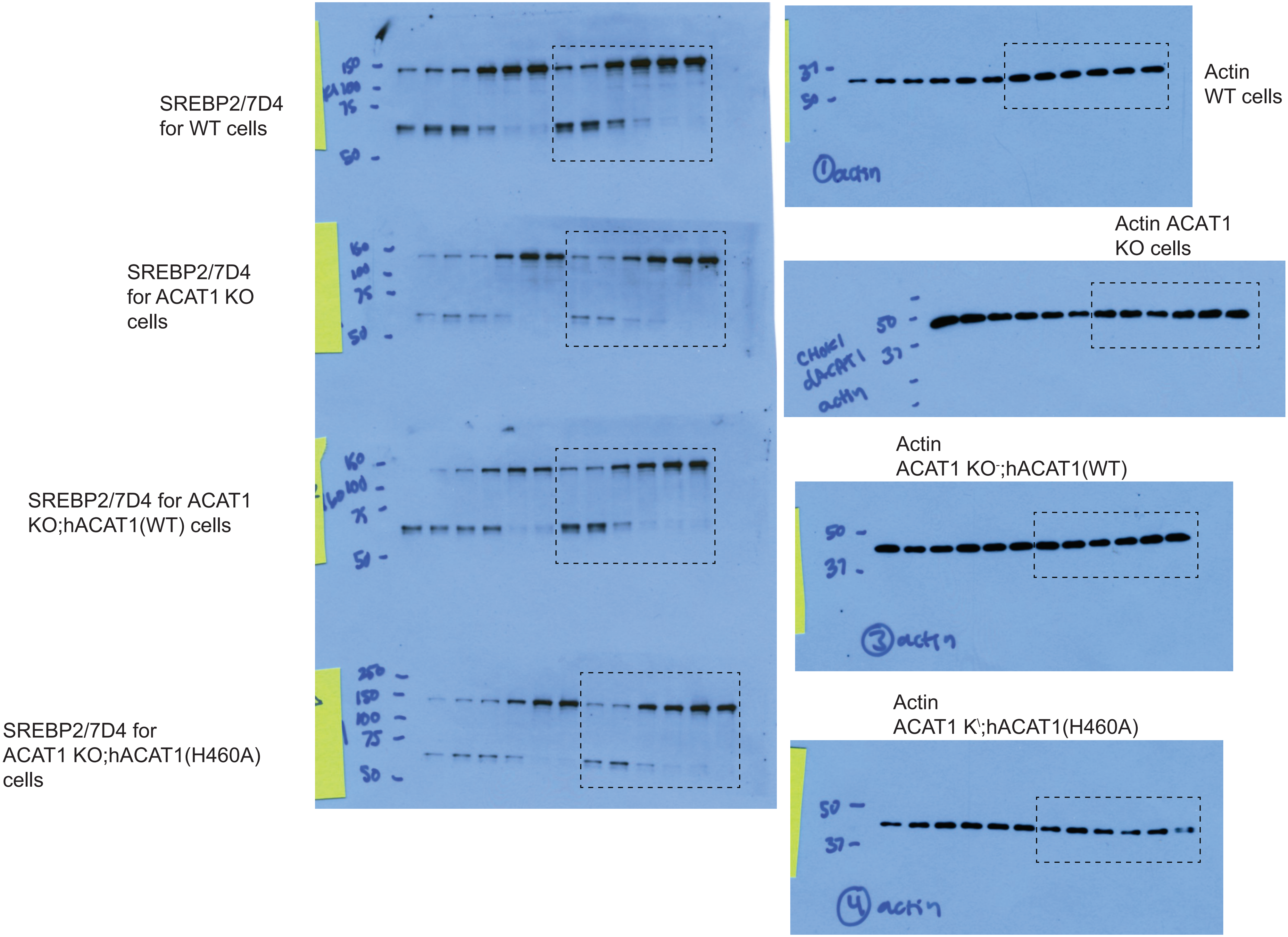

Supplement: Figure 2—figure supplement 3—source data 1. [file elife-83534-fig2-figsupp3-data1.zip › Figure 2-figure supplement 3-source data 1/Figure 2-figure supplement 3-source data 1.pdf]
