# Supplementary material for: A concerted mechanism involving ACAT and SREBPs by which oxysterols deplete accessible cholesterol to restrict microbial infection
Source: eLife. 2023 Jan 25;12:e83534. doi: 10.7554/eLife.83534 (PMC9925056; doi:10.7554/eLife.83534)
Supplement: Figure 4—source data 1. [file elife-83534-fig4-data1.zip › Figure 4-source data 1/Figure 4-souce data 1.pdf]

Figure 4 - Source Blots

A

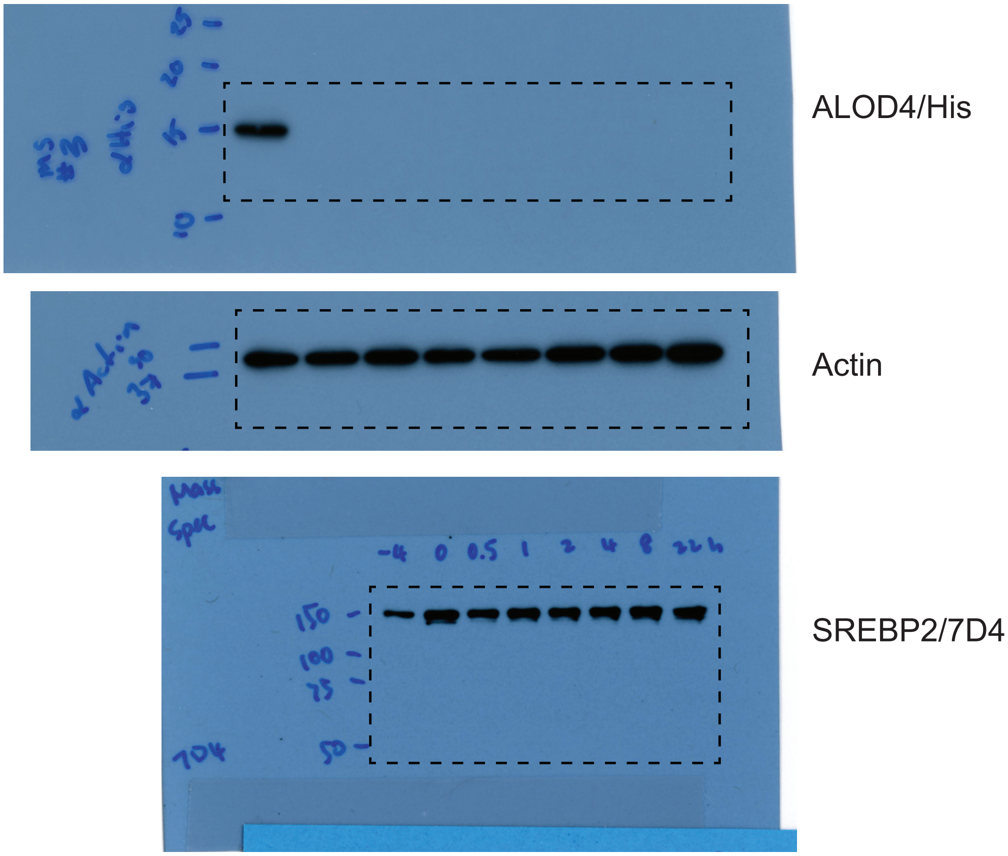

B

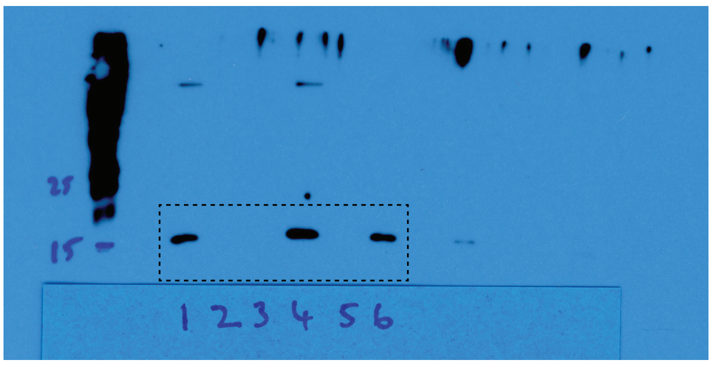

ALOD4/His for N-BP1a cells

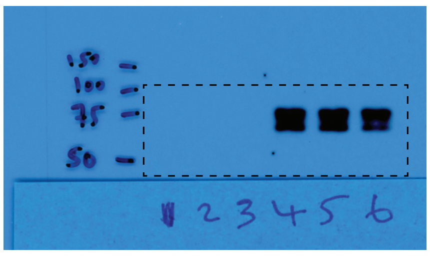

N-BP1a/FLAG

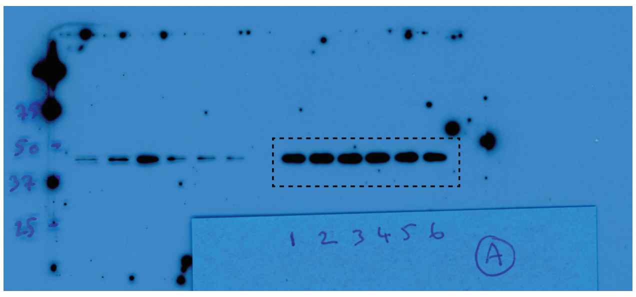

Actin for N-BP1a cells

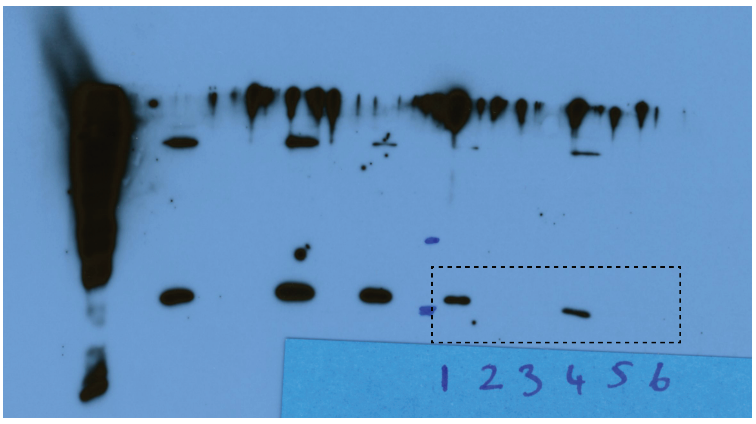

ALOD4/His for N-BP1c cells

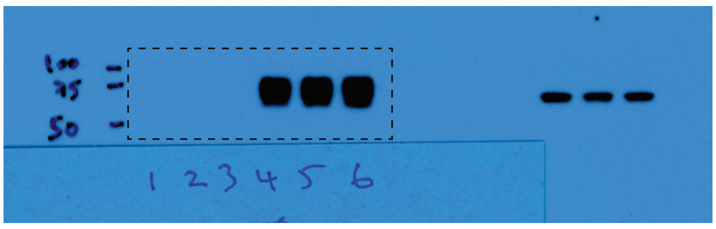

N-BP1c/FLAG

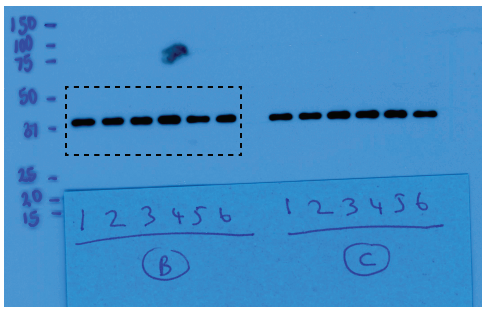

Actin for N-BP1c cells

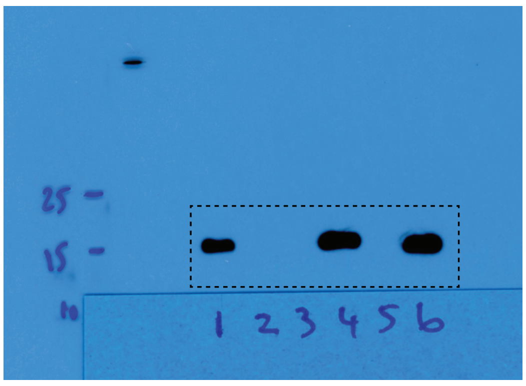

ALOD4/His for N-BP2 cells

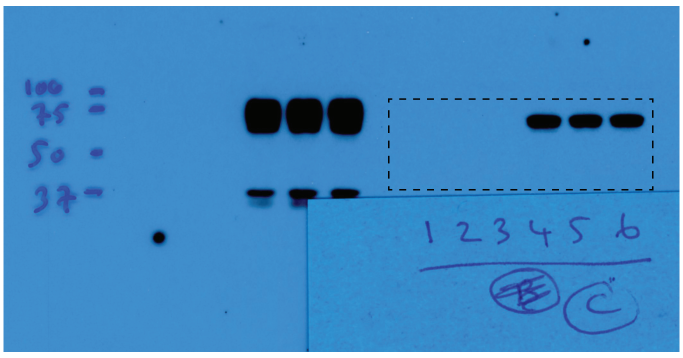

N-BP2/FLAG

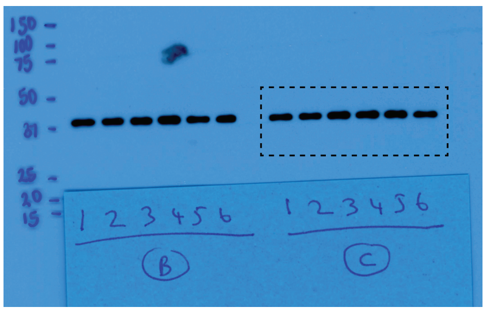

Actin for N-BP2 cells

C

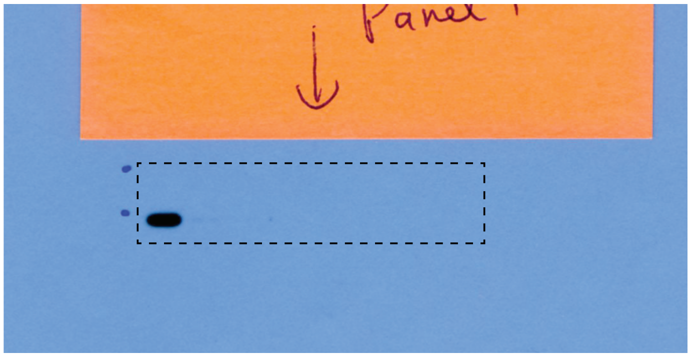

ALOD4/His for None

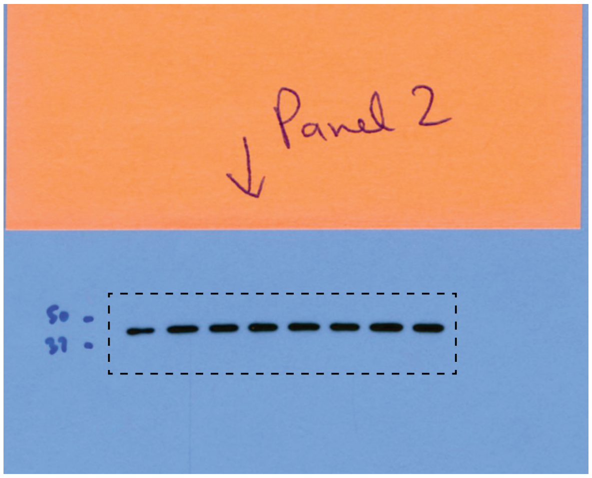

Actin for None

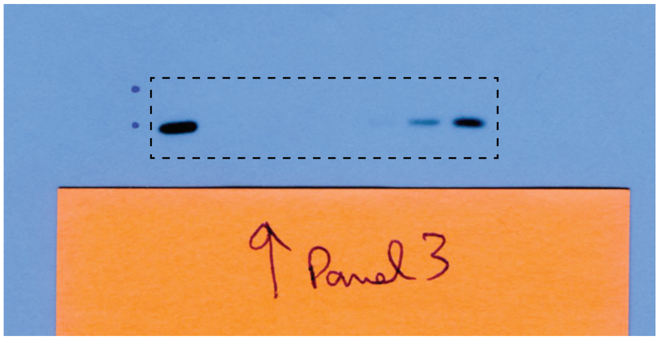

ALOD4/His for 10 uM Inhibitor

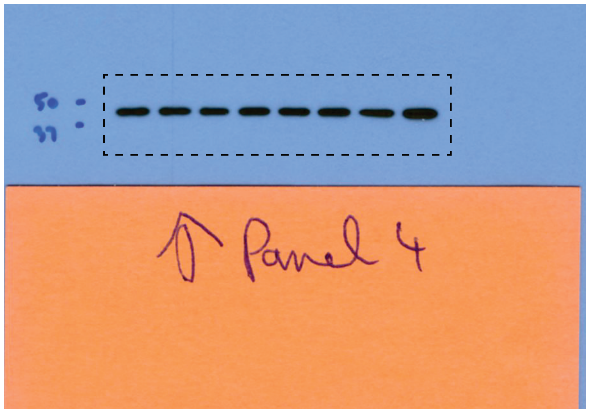

Actin for 10 uM Inhibitor

D

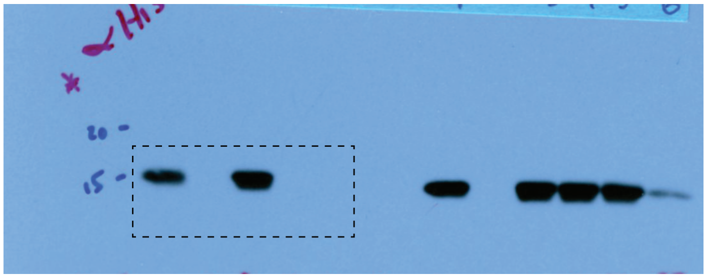

ALOD4/His for WT cells

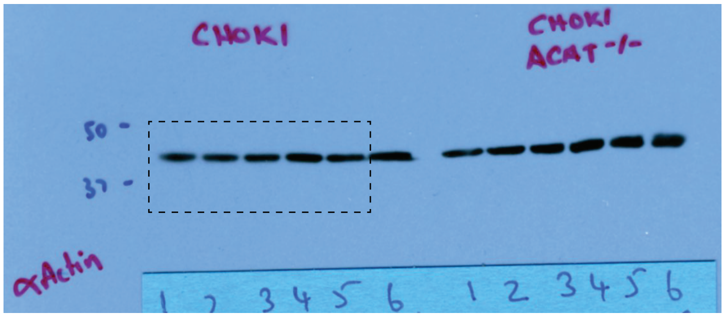

Actin for WT cells

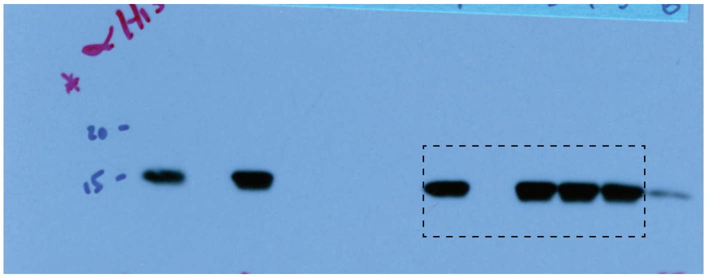

ALOD4/His for ACAT1 KO cells

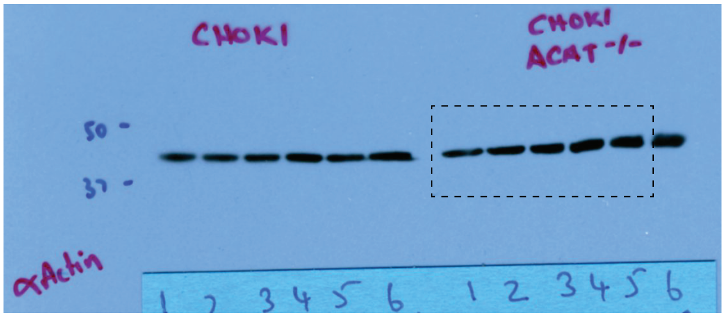

Actin for ACAT1 KO cells
