# Supplementary material for: A concerted mechanism involving ACAT and SREBPs by which oxysterols deplete accessible cholesterol to restrict microbial infection
Source: eLife. 2023 Jan 25;12:e83534. doi: 10.7554/eLife.83534 (PMC9925056; doi:10.7554/eLife.83534)
Supplement: Figure 7—figure supplement 1—source data 1. [file elife-83534-fig7-figsupp1-data1.zip › Figure 7-figure supplement 1-source data 1/Figure 7-figure supplement 1-source data 1.pdf]

Figure 7 figure supplement 1 - Source Blots

C

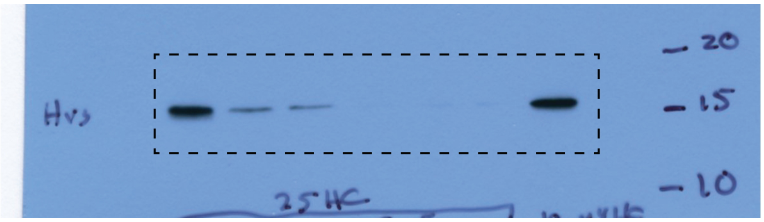

ALOD4/His
for Huh7.5 cells

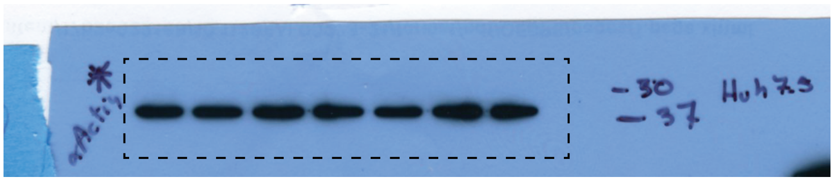

Actin for
Huh7.5 cells

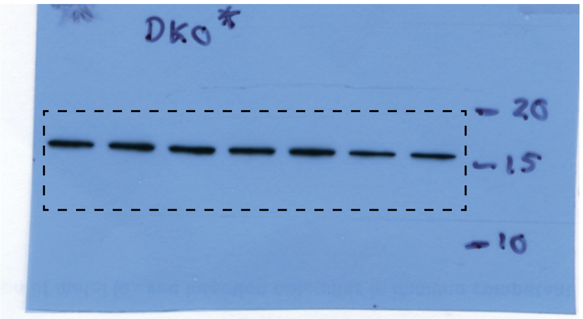

ALOD4/His
for Huh7.5$^{\Delta ACAT}$ cells

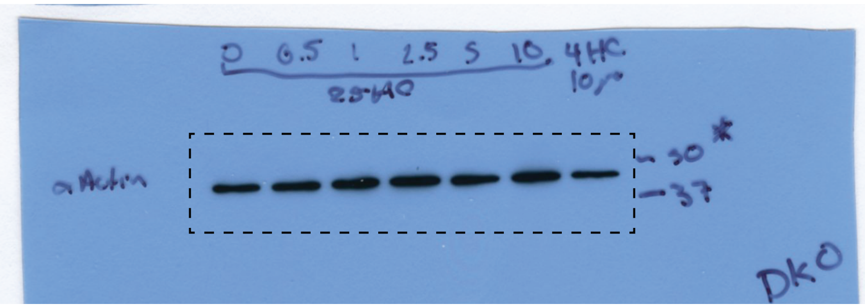

Actin for
Huh7.5$^{\Delta ACAT}$ cells
